# Safety and Short-term Outcomes of High-Dose Erythropoietin in Preterm Infants With Intraventricular Hemorrhage: The EpoRepair Randomized Clinical Trial

**DOI:** 10.1001/jamanetworkopen.2022.44744

**Published:** 2022-12-02

**Authors:** Sven Wellmann, Cornelia F. Hagmann, Stefanie von Felten, Leonard Held, Katrin Klebermass-Schrehof, Anita C. Truttmann, Claudia Knöpfli, Jean-Claude Fauchère, Christoph Bührer, Hans Ulrich Bucher, Christoph M. Rüegger

**Affiliations:** 1 Department of Neonatology, University Children’s Hospital Regensburg, Hospital St Hedwig of the Order of St John, University of Regensburg, Regensburg, Germany; 2 Division of Neonatology, University Children’s Hospital Basel, University of Basel, Basel, Switzerland; 3 Department of Neonatology and Pediatric Intensive Care, University Children’s Hospital Zurich, Zurich, Switzerland; 4 Children’s Research Center, University Children’s Hospital Zurich, Zurich, Switzerland; 5 Department of Biostatistics, Institute of Epidemiology, Biostatistics and Prevention, University of Zurich, Zurich, Switzerland; 6 Department of Pediatrics and Adolescent Medicine, Medical University Vienna, Vienna, Austria; 7 Clinic of Neonatology, Department of Women Mother Child, University Center Hospital and University of Lausanne, Vaud, Switzerland; 8 Newborn Research, Department of Neonatology, University Hospital Zurich, University of Zurich, Zurich, Switzerland; 9 Department of Neonatology, Charité–Universitätsmedizin Berlin, Berlin, Germany

## Abstract

**Importance:**

Intraventricular hemorrhage (IVH) is a major cause of neonatal morbidity and mortality in preterm infants without a specific medical treatment to date.

**Objective:**

To assess the safety and short-term outcomes of high-dose erythropoietin in preterm infants with IVH.

**Design, Setting, and Participants:**

Between April 1, 2014, and August 3, 2018, a randomized double-blind clinical trial enrolled 121 preterm infants (gestational age <32 weeks or birth weight <1500 g) aged 8 or less days with moderate to severe IVH identified by cerebral ultrasonography from 8 Swiss and Austrian tertiary neonatal units. Statistical analyses were performed between October 1, 2019, and September 12, 2022.

**Interventions:**

Infants received intravenous high-dose erythropoietin (2000 units/kg body weight) or placebo at 4 time points between weeks 1 and 4 of life.

**Main Outcomes and Measures:**

Secondary outcomes included (1) mortality and morbidity rates and (2) brain magnetic resonance imaging findings at term-equivalent age (TEA). The primary outcome was the composite intelligence quotient at 5 years of age (not available before 2023).

**Results:**

Sixty infants (48% male [n = 29]) were randomly assigned to receive erythropoietin, and 61 infants (61% male [n = 37]) were randomly assigned to receive placebo. The median birth weight was 832 g (IQR, 687-990 g) in the erythropoietin group and 870 g (IQR, 680-1110 g) in the placebo group. Median gestation was 26.1 weeks (IQR, 24.8-27.3 weeks) in the erythropoietin group and 27.0 weeks (24.9–28.1 weeks) in the placebo group. The 2 groups had similar baseline characteristics and morbidities. Up to TEA, 10 newborns died (16.7%) in the erythropoietin group, and 5 newborns (8.2%) died in the placebo group (adjusted odds ratio, 2.24 [95% CI, 0.74-7.66]; *P* = .15). Infants receiving erythropoietin had higher mean hematocrit levels. Conventional magnetic resonance imaging at TEA for 100 infants showed no significant differences in global or regional brain injury scores.

**Conclusions and Relevance:**

This preliminary report of a randomized clinical trial found no evidence that high-dose erythropoietin in preterm infants with IVH affects brain injury scores on conventional magnetic resonance imaging at TEA. Higher mortality in the erythropoietin group was not significant but should be reassessed based on future results from similar trials.

**Trial Registration:**

ClinicalTrials.gov Identifier: NCT02076373

## Key Points

**Question:**

Does treatment with high-dose, intravenous recombinant human erythropoietin for preterm infants with moderate to severe intraventricular hemorrhage (IVH) improve short- and long-term neurological outcomes?

**Findings:**

In this preliminary report of a randomized, double-blind clinical trial including 121 preterm infants with moderate to severe IVH, treatment with intravenous high-dose erythropoietin did not significantly change short-term outcomes or brain injury scores assessed by cranial magnetic resonance imaging at term-equivalent age.

**Meaning:**

In preterm infants with moderate to severe IVH, high-dose erythropoietin had no significant treatment effect up to term-equivalent age; whether a treatment effect on long-term neurological development can be observed remains to be seen.

## Teaser

This preliminary report of a randomized clinical trial assess the safety and short-term outcomes of high-dose erythropoietin in preterm infants with intraventricular hemorrhage.

## Introduction

About one-quarter of very preterm infants (gestational age <32 weeks) have intraventricular hemorrhage (IVH), a major cause of neonatal morbidity and mortality.^1^ Survivors of IVH are at increased risk of neurodevelopmental impairment later in life.^2^ Intraventricular hemorrhage typically results from the rupture of a blood vessel in the germinal matrix linked to hemodynamic instability, infection, and other stressors around birth.^3^ Primary brain damage from IVH as well as secondary injury due to inflammation or posthemorrhagic hydrocephalus (PHH) contribute to the complex cerebral disease of IVH, for which no treatment is yet available.^4^

Observational data on preterm infants with IVH treated with recombinant human erythropoietin to prevent red blood cell transfusion showed improved long-term neurodevelopmental outcomes when assessed beyond 3 years of age^5^ compared with controls. Because they received erythropoietin several days or weeks after the insult, its proposed mode of action is thought to be the facilitation of repair, sustaining neuronal growth, and differentiation after brain injury rather than protection against damage itself.^6^ Meta-analysis of randomized clinical trials with prophylactic erythropoietin showed improved cognitive development in very preterm infants assessed at a corrected age of 18 to 24 months,^7^ although this effect was completely abolished if 1 study with a high risk of bias was excluded. Reassuringly, however, cumulative data from all trials of low- and high-dose erythropoietin in preterm infants confirmed product safety.^8^

The current Erythropoietin for the Repair of Cerebral Injury in Very Preterm Infants (EpoRepair) trial is investigating the safety and efficacy of high-dose erythropoietin after a diagnosis of moderate to severe IVH in preterm infants.^9^ The present preliminary report covers the clinical course to hospital discharge and characterization of the specific features of encephalopathy of prematurity, including the structural changes in white and gray matter detected on magnetic resonance imaging (MRI) scans at term-equivalent age (TEA).

## Methods

### Study Patients

**Figure 1.**
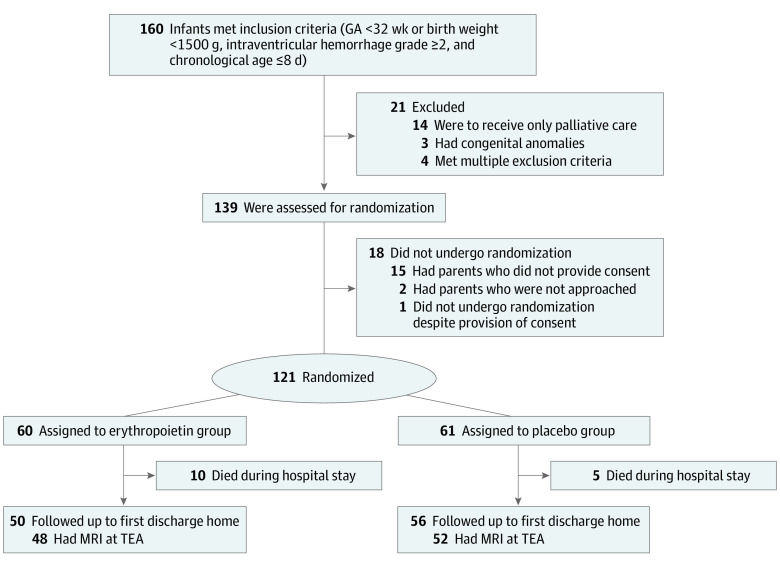
Flow Diagram GA indicates gestational age; MRI, magnetic resonance imaging; and TEA, term-equivalent age.

Infants were eligible if born at a gestational age younger than 32 completed weeks or birth weight less than 1500 g who received a diagnosis at the chronological age of 8 or less days of IVH with a grade of 2 or higher.^10^ Exclusion criteria were known life-threatening anomalies, chromosomal anomalies, or known intrauterine viral or protozoic infection. This study followed the Consolidated Standards of Reporting Trials (CONSORT) reporting guideline (Figure 1) and was conducted at 8 Swiss and Austrian tertiary neonatal units. Here we present the preliminary report of this study.

### Study Oversight

The trial was approved by the research ethics boards at the University of Zurich in Zurich, Switzerland, and by each participating center in Switzerland University Hospital Zurich, University Children’s Hospital Basel, Kantonsspital Aarau, Inselspital Bern, Kantonsspital Chur, University Center Hospital and University of Lausanne, Kantonsspital St Gallen, and Austria Medical University of Vienna and is registered in the European Union Drug Regulating Authorities Clinical Trials Register (EudraCT 2014-000612-34) and at ClincalTrials.gov (NCT02076373). Appropriate regulatory approvals and written informed consent from parents or guardians were obtained before randomization. All authors vouch for the accuracy and completeness of the data and the fidelity of the report to the trial protocol (see the trial protocol in Supplement 1), which was previously published.^9^ Three hospital pharmacies prepared the study drug for all participating centers (Bern and Zurich for Switzerland; Vienna for Austria); the verum, recombinant human Epoetin beta, was purchased from Roche.

### Randomization

A block-randomization scheme with a fixed block size of 4 was used to assign infants in a 1:1 ratio to erythropoietin or placebo, stratified by participating center. The 3 hospital pharmacies received the sequence of study drug assignments from a statistician at the coordinating center in Zurich (clinical trial unit Zurich) and prepared the study medication on request per patient and prescription.

### Study Medication

After enrollment, the initial erythropoietin or placebo dose had to be administered before day 9 of life. Infants received erythropoietin at a dose of 2000 U/kg body weight or placebo (saline) intravenously every 24 hours for 3 subsequent days; thereafter, a fourth dose was administered 10 days after the initial dose and, finally, a fifth dose 17 days after the initial dose. With the exceptions of the data coordinating center staff and site pharmacist, all trial personnel, including those administering the injections, were unaware of the trial group assignments. Additional protocol details have been published previously.^9^ All other interventions were prescribed at the discretion of the local clinicians.

### Primary Outcome

The primary end point was the composite intelligence quotient at 5 years of age. This outcome will be determined using the Kaufmann Assessment Battery for Children,^11^ and it will be reported together with the overall developmental outcome assessed by neurological and formal psychological examination after study completion.

### Secondary Outcomes

The key secondary outcome comprises a global brain abnormality (GBA) score and white and gray matter injury scores assessed on T1- and T2-weighted MRI images at TEA on 3-T magnetic resonance systems (Siemens Medical Solutions) at the treatment sites. For MRI scoring, we applied a previously established approach^12^ used with modifications elsewhere.^13,14^ In brief, the GBA score consists of 40 points from normal (0 points) to severely abnormal (40 points) and is classified as normal or mild vs moderate or severe according to Kidokoro et al.^14^ It comprises 4 subscores. First, the white matter injury score was obtained by adding the subscores of white matter signal abnormality (the so-called diffuse excessive high signal intensity), periventricular white matter volume loss, presence of cystic abnormalities, ventricular dilatation, and thinning of corpus callosum (continuous 0-17, binary normal or mild vs moderate or severe). Second, the gray matter injury score was obtained by adding the subscores of cortical abnormalities, quality of gyral maturation, and size of subarachnoid space (continuous 0-9, binary normal or mild vs moderate or severe). Third, the subscore for deep gray matter abnormality (continuous 0-7, binary normal or mild vs moderate or severe) and the subscore for cerebellar abnormality (continuous 0-7, binary normal or mild vs moderate or severe) completed the GBA score.

Other prespecified secondary outcomes, all of them occurring after study dosing and up to TEA, were the following: death for any reason; clinical course of IVH, including PHH; bronchopulmonary dysplasia (BPD) in survivors at 36 weeks’ postmenstrual age (PMA), as defined previously^15^; retinopathy of prematurity (ROP; international classification stage ≥2 or requiring treatment); culture-proven infections (defined as episodes of sepsis or meningitis confirmed by blood or cerebrospinal fluid culture growing bacteria, fungi, or viruses); patent ductus arteriosus (PDA) requiring drug treatment or surgery; focal intestinal perforation (FIP) or necrotizing enterocolitis (NEC; diagnosed during surgery, at autopsy, or by a finding of pneumatosis intestinalis, hepatobiliary gas, or free intraperitoneal air on abdominal radiograph); and length of hospital stay (LOS).

### Sample Size

A formal sample size calculation was performed with regard to the primary outcome at 5 years of age. Based on data from Neubauer et al^5^ and some adaptations to our study population,^9^ we hypothesized a mean (SD) difference of 10 (15) IQ points between the erythropoietin and placebo groups. To achieve 80% power at a significance level of 5%, a total sample size of 72 is required (36 per group). In this highly vulnerable population, we expected an early patient loss of 15% up to TEA, compounded by a further 20% loss to follow-up between TEA and 5-year follow-up. Hence, we decided to recruit 120 patients to achieve 100 MRI scans at TEA and to have 80 patients with 5-year follow-up.

### Statistical Analysis

Statistical analysis was planned in advance^9^ and sealed in June 2019 (see the statistical analysis plan in Supplement 1). All analyses were performed using R, version 4.2.1 (R Project for Statistical Computing).^16^

Demographic and baseline characteristics as well as all outcomes were summarized per trial group for the full analysis set. Mean (SD) values (or, if more appropriate, median values and IQRs) were reported for continuous variables. Frequencies and percentages are reported for categorical variables. The *z* scores and percentiles for birth weight and head circumference as well as the categorizations of small for gestational age (<10th percentile for weight), large for gestational age (>90th percentile for weight), or appropriate for gestational age (between 10th and 90th percentile for weight) were calculated using the electronic growth chart calculator based on preterm growth charts.^17,18^

Outcomes were compared between groups using linear or generalized linear mixed-effects models (the latter with binomial error and logit link), with treatment as fixed explanatory factor and a random intercept per “center.” The resulting estimates of erythropoietin treatment effect (mean differences for continuous outcomes and odds ratios for binary outcomes) are adjusted for center effects and reported together with 95% CIs (applying a significance level of 5%) and 2-sided *P* values. In some cases, we had to switch from a mixed-effects model to a fixed-effects model without center effects because either the random-effects variance could not be estimated (continuous outcome cerebral white matter abnormality) or because of a singular model fit (PHH with drainage at 28 days of life and PMA of 36 weeks, NEC, or FIP). For those outcomes, we report *P* values and 95% CIs from a standard *z* test. LOS was analyzed as time to hospital discharge (alive) using a cause-specific Cox proportional hazards regression model, accounting for the competing risk of in-hospital death by censoring patients at time of death (recommended by Brock et al^19^). Treatment and center were included as fixed effects.

For all outcomes, a post hoc sensitivity analysis accounting for the nonindependence of twins was performed, with a random intercept per family nested in center. Aggregate trial data on mortality were compared using R.

## Results

### Study Patients

Between April 1, 2014, and August 3, 2018, 121 infants were randomly assigned from 8 study centers in Switzerland and Austria (Figure 1). They included 7 pairs of twins, all but 1 of whom were randomly assigned to different groups. Infant and maternal baseline characteristics were similar in both groups (Table 1).

**Table 1.** Baseline Characteristics of Mothers and Infants

Characteristic	No. (%)	
	Erythropoietin group (n = 60)	Placebo group (n = 61)
**Mothers**		
Age, median (IQR), y	34.0 (30.0-37.0)	33.0 (29.8-37.0)
Pregnancy first	31 (51.7)	38 (62.3)
Parturition first	26 (43.3)	40 (65.6)
PPROM	16 (26.7)	17 (27.9)
Chorioamnionitis	19 (31.7)	18 (29.5)
Preeclampsia	9 (15.0)	9 (14.8)
Use of antenatal steroids	52 (86.7)	57 (93.4)
Cesarean section	53 (88.0)	42 (68.9)
**Infants**		
Gestational age, median (IQR), wk	26.1 (24.8-27.3)	27.0 (24.9-28.1)
Birth weight, median (IQR), g	832 (687-990)	870 (680-1110)
Sex		
Male	29 (48.3)	37 (60.7)
Female	31 (51.7)	24 (39.3)
Singleton birth	33 (55.0)	41 (67.2)
Umbilical cord pH, mean (SD)	7.30 (0.13)	7.28 (0.08)
Apgar score, median (IQR)		
At 5 min	6.0 (5.0-8.0)	7.0 (5.0-8.0)
At 10 min	8.0 (6.0-9.0)	8.0 (6.0-9.0)
CRIB score, median (IQR)	6.0 (2.3-8.0)	6.0 (1.8-9.0)
Intraventricular hemorrhage		
Grade 2	27 (45.0)	23 (37.7)
Grade 3	10 (16.7)	17 (27.9)
Grade 4	23 (38.3)	21 (34.4)

Abbreviations: CRIB, Clinical Risk Index for Babies; PPROM, preterm premature rupture of membranes (>18 hours before delivery).

### Clinical Outcome

Fifteen of the 121 infants died before TEA, and 1 infant died after TEA. The remaining 105 infants were discharged home, 100 of whom underwent MRI at TEA. There was higher mortality up to TEA with erythropoietin (n = 10 [16.7%]) compared with placebo (n = 5 [8.2%]), yet the difference was nonsignificant (odds ratio, 2.24 [95% CI, 0.74-7.66]; *P* = .15; relative risk, 2.03 [95% CI, 0.74-5.60]). Kaplan-Meier survival curves demonstrate the occurrence of all deaths except 1 within 5 to 20 days of life and all at similar gestational age in both study groups (eFigure 1 and eFigure 2 in Supplement 2). No single cause of death recorded on a death certificate or autopsy report explained the difference in mortality between the 2 groups.

**Figure 2.**
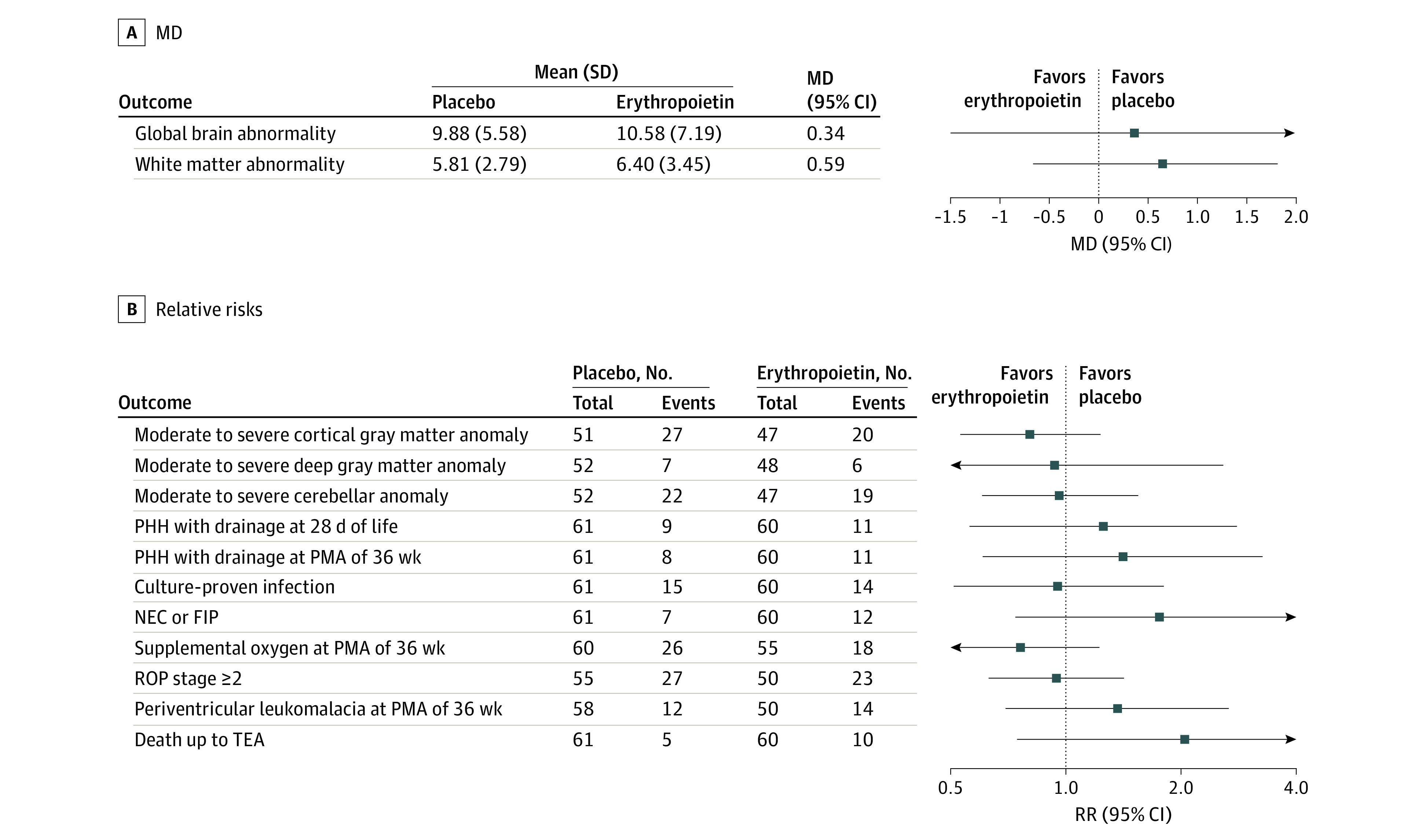
Forest Plots for Mean Differences (MDs) and Relative Risks (RRs) of Short-term Outcomes Until Term-Equivalent Age (TEA) and Magnetic Resonance Imaging Scores at TEA FIP indicates focal intestinal perforation; NEC, necrotizing enterocolitis; PHH, posthemorrhagic hydrocephalus; PMA, postmenstrual age; and ROP, retinopathy of prematurity.

Nor did the study groups differ significantly in the incidence of other prespecified outcomes, including infections, ROP, BPD, NEC or FIP, medically treated PDA, or LOS (Figure 2; eTable in Supplement 2). There was also no significant difference in the frequencies of clinical findings directly related to the course of IVH, including PHH, need for transient or permanent cerebrospinal fluid drainage, or diffuse and cystic periventricular leukomalacia (PVL) (Figure 2; eTable in Supplement 2). There was only a trend toward longer LOS of patients in the erythropoietin group, with a median LOS of 91 days (95% CI, 85-105 days), compared with the placebo group (median LOS, 85 days [95% CI, 80-99 days]) (eTable in Supplement 2).

In contrast, infants who received erythropoietin had a higher mean (SD) hematocrit level of 34.30 [6.08] [to convert hematocrit to a proportion of 1.0, multiply by 0.01]) than infants who received placebo at 36 weeks’ PMA (32.59% [4.88%]). Adjusting for baseline hematocrit level, gestational age, IVH degree, and median number of erythrocyte transfusions (3 [IQR, 1-6] in the erythropoietin group and 3 [IQR, 0-5] in the placebo group), we estimated an increase in the hematocrit level of 2.12% (95% CI, 0.24%-3.98%; *P* = .03) with erythropoietin compared with placebo (Table 2). Results of the sensitivity analysis accounting for the nonindependence of twins were very similar to the results shown here.

**Table 2.** Coefficient Estimates for Treatment With Erythropoietin vs Placebo as Well as Several Covariates From a Mixed-Effect Model on Hematocrit Levels Determined at 36 Weeks’ Postmenstrual Age

Variable	Coefficient (95% CI)	*t* Value	*P* value
Hematocrit level at first day of life	0.06 (−0.07 to 0.19)	0.88	.38
Gestational age at birth	0.02 (−0.51 to 0.54)	0.07	.94
IVH grade			
3 (vs 2)	0.001 (2.57 to 2.52)	0.001	>.99
4 (vs 2)	−0.18 (−2.27 to 1.91)	−0.17	.87
No. of red blood cell transfusions	0.34 (0.09 to 0.59)	2.61	.01
Treatment with erythropoietin	2.12 (0.24 to 3.98)^a^	2.18	.03

Abbreviation: IVH, intraventricular hemorrhage.

^a^ The coefficient for treatment with erythropoietin corresponds to an adjusted estimate for the effect of erythropoietin vs placebo. Further, the model estimates that an additional red blood cell transfusion is associated with higher levels of hematocrit (increased by 0.34).

### MRI Outcome

Although empirical cumulative distributions of GBA scores indicated a trend toward less brain abnormality in the erythropoietin group (eFigure 3 in Supplement 2), there was no significant treatment effect in any MRI outcome (Figure 2; eFigure 4 and eTable in Supplement 2). Further, in exploratory analyses, we assessed the association of certain clinical risk factors with the main MRI outcome of GBA score together with treatment. Only pretreatment-assessed IVH grade and PHH with drainage at 36 weeks’ PMA were significantly associated with a poorer GBA score (Table 3).

**Table 3.** Coefficient Estimates for Treatment With Erythropoietin vs Placebo as Well as Several Covariates From a Mixed-Effect Model on Secondary Outcome of Global Brain Abnormality Score Determined by Magnetic Resonance Imaging at Term-Equivalent Age

Variable	Coefficient (95% CI)	*t* Value	*P* value
Gestational age at birth	−0.46 (−0.94 to 0.02)	−1.91	.06
IVH grade			
3 (vs 2)	1.37 (−1.36 to 4.10)	0.10	.32
4 (vs 2)	3.04 (0.87 to 5.22)	2.78	.007
PHH with drainage at 36 wk PMA	9.01 (6.33 to 11.70)	6.66	<.001
Supplemental oxygen at 36 wk PMA	1.36 (−0.71 to 3.43)	1.30	.20
Sepsis	−0.38 (−2.74 to 1.98)	−0.32	.75
Treatment with erythropoietin	0.34 (−1.57 to 2.25)^a^	0.35	.72

Abbreviations: IVH, intraventricular hemorrhage; PHH, posthemorrhagic hydrocephalus; PMA, postmenstrual age.

^a^ The coefficient for treatment with erythropoietin corresponds to an adjusted estimate for the effect of erythropoietin vs placebo. Further, the model estimates that IVH grade 4 (vs 2) and PHH with drainage at 36 weeks’ PMA are associated with higher values of global brain abnormality (increase by 3.04 and 9.01, respectively).

## Discussion

We report intermediate safety and outcome data from of the EpoRepair trial, a multinational, prospective, and randomized double-blind clinical trial of intravenous high-dose erythropoietin vs placebo in preterm infants with confirmed moderate to severe IVH. The primary outcome, due for completion in 2023, is cognitive improvement at 5 years of age.^9^ Key findings are that there were no significant differences up to TEA in complications associated either with preterm birth in general (sepsis, ROP, BPD, NEC, PDA, and PVL) or with IVH in particular (cerebral infarction and PHH), that there were no significant differences between patients who received erythropoietin and patients who received placebo in brain injury scores assessed by cranial MRI at TEA, and that there was a significant effect of treatment with erythropoietin on hematopoiesis with an increase in hematocrit level.

Brain injury MRI scores at TEA are a secondary end point for which the trial was not powered. However, in an exploratory analysis, we assessed the association of certain risk factors with the main MRI outcome, GBA score, together with the study treatment. The results suggest that severe (grade 4) IVH and the presence of PHH with drainage are most strongly associated with GBA. Gestational age at birth, sepsis or oxygen dependency at 36 weeks’ PMA, and study treatment were not associated with GBA.

In contrast to the previous trial^13^ assessing the neuroprotective effect of early high-dose erythropoietin in the first 42 hours after birth in preterm infants born before 32 weeks, which reported less MRI brain abnormality than with placebo, the EpoRepair trial includes fewer scanned patients (100 vs 165), all with grade 2 or higher IVH, and several with subsequent complications, such as PHH and PVL. The earlier trial excluded infants with grade 2 or higher IVH detected within the first 36 hours of life. In line with our findings, brain MRI data from the large, US Preterm Erythropoietin Neuroprotection Trial (PENUT)^20,21^ show no difference between the erythropoietin and placebo groups.

Low-dose erythropoietin (200-400 U/kg body weight, usually administered 3 times per week) is widely used to stimulate hematopoiesis in preterm infants and is considered safe and effective when given early or late after birth.^8,22^ Moderate- to high-dose erythropoietin (500-3000 U/kg body weight) was found to be safe for use as a neuroprotective agent in 3 large, prospective trials enrolling preterm infants at birth.^23,24,25^ The Swiss trial^25^ reported a 5.2% (12 of 229) mortality rate in the erythropoietin group vs a 5.6% (12 of 214) mortality rate in the placebo group. The Chinese trial^23^ reported a mortality rate of 6.4% (21 of 330) in the erythropoietin group vs 10.1% (34 of 338) in the control group. The US PENUT^24^ reported a mortality rate of 13.2% (63 of 476) in the erythropoietin group vs 10.9% (50 of 460) in the placebo group. The mortality rates in the EpoRepair trial are 16.7% (10 of 60) in the erythropoietin group vs 8.2% (5 of 61) in the placebo group. Higher mortality in PENUT and the EpoRepair trial may reflect the inclusion of more immature infants (mean gestational age, 25.9 weeks in PENUT and 26.6 weeks in the EpoRepair trial vs 29.1 weeks in the Swiss trial and 30.4 weeks in the Chinese trial). Analysis of the aggregate PENUT and EpoRepair data shows no significant difference in mortality (73 of 537 patients [13.6%] in the erythropoietin group vs 55 of 521 patients [10.6%] in the placebo group; odds ratio, 1.33 [95% CI, 0.92-1.94]; *P* = .13).

### Limitations

This study has limitations. First, while the multicenter setting has the advantage that the results are more generalizable, it may have introduced more heterogeneity to our relatively small trial. In addition, enrollment of multiples was not anticipated, and thus accounting for them was not considered in the statistical analysis plan. However, results of post hoc sensitivity analyses were very similar to those of the prespecified analyses.

## Conclusions

In conclusion, the present preliminary outcome report from the multinational, prospective, randomized, double-blind, placebo-controlled EpoRepair trial of high-dose intravenous erythropoietin in preterms with demonstrated moderate to severe IVH confirms the absence of treatment effect on brain imaging and clinical end points up to TEA. To what extent a treatment effect on neurological development can be observed at 5 years of age remains to be seen.

## Notes

Supplement 1.
Trial Protocol and Statistical Analysis Plan

Supplement 2. **eFigure 1.** Kaplan-Meier Curves for Overall Survival After Birth in the Erythropoietin (Epo) and Placebo Group in Regard to Days of Life **eFigure 2.** Kaplan-Meier Curves for Overall Survival After Birth in the Erythropoietin (Epo) and Placebo Group in Regard to Postmenstrual Age (Weeks) **eTable.** Secondary Outcomes Up to Term Equivalent Age or Hospital Discharge **eFigure 3.** Empirical Cumulative Distribution of Global Brain Abnormality Scores in the Erythropoietin (Epo) and Placebo Group **eFigure 4.** Forest Plot of Odds Ratio (OR) Estimates (Boxes) and 95% Confidence Intervals (Lines) of the Erythropoietin (Epo) vs Placebo Treatment Effect on All Binary Secondary Outcomes Shown in the eTable of the Supplement File

Supplement 3.
Nonauthor Collaborators

Supplement 4.
Data Sharing Statement
