# Supplementary material for: Safety and Short-term Outcomes of High-Dose Erythropoietin in Preterm Infants With Intraventricular Hemorrhage: The EpoRepair Randomized Clinical Trial
Source: JAMA Netw Open. 2022 Dec 2;5(12):e2244744. doi: 10.1001/jamanetworkopen.2022.44744 (PMC9719050; doi:10.1001/jamanetworkopen.2022.44744)
Supplement: Supplement 1. — Trial Protocol and Statistical Analysis Plan [file jamanetwopen-e2244744-s001.pdf]

Clinical Study Protocol

Title: Erythropoietin for the repair of cerebral injury in very preterm infants

- a randomized, double-blind, placebo-controlled, prospective, and multicentre clinical study

Short title: EpoRepair

Sponsor and Principal Investigator:

Sponsor:
Prof. Dr. med. Hans Ulrich Bucher
Weiterbildungsbeauftragter, ärztliche Direktion
UniversitätsSpital Zürich, 8091 Zürich
Phone +41 44 255 5340, Fax +41 44 255 4442,
Email buh@usz.ch

Coordinating Principal Investigator:
Prof. Dr. med. Sven Wellmann
Universitäts- Kinderspital beider Basel (UKBB)
4056 Basel
Phone +41 61 7042924, Fax +41 61 255 5956,
Email sven.wellmann@ukbb.ch

Study Product: Erythropoietin (Recormon)

Protocol Version and Date: Version 1.11, March 20, 2017

CONFIDENTIAL

The information contained in this document is confidential and the property of the department of Neonatology of the University Hospital Zurich. The information may not - in full or in part - be transmitted, reproduced, published, or disclosed to others than the applicable Independent Ethics Committee(s) and Regulatory Authority(ies) without prior written authorization from the department of Neonatology of the University Hospital Zurich, except to the extent necessary to obtain informed consent from those subjects who will participate in the study.

STUDY SYNOPSIS

**Sponsor**	Prof. Dr. med. Hans Ulrich Bucher
**Study Title:**	Erythropoietin for the repair of cerebral injury in very preterm infants
**Short Title/Study ID:**	EpoRepair
**Protocol Version and Date:**	Version number 1.11, March 20, 2017
**Clinical Phase:**	Phase 3
**Methodology:**	Randomized (verum:placebo = 1:1), double-blind, placebo-controlled, prospective, and multicentre clinical study
**Study Duration:**	After 3 weeks of study treatment, 2-4 months until last study-specific procedure, followed by a 5 year follow-up.
**Study Centre(s):**	Multi-centre, 7 centres in Switzerland, 1 in Germany, 1 in Austria.
**Coordinating Principal Investigator:**	Prof. Dr. med. Sven Wellmann<br>Universitäts- Kinderspital beider Basel (UKBB), 4056 Basel<br>Phone +41 61 7042924, Fax +41 61 255 5956,<br>Email sven.wellmann@ukbb.ch
**Objective(s)/ Outcome(s):**	Primary objective: At 5 years of age: Intelligence quotient (Kaufman Assessment Battery for Children, German version (K-ABC)<br>Secondary objectives:<br>1) cerebral ultrasound (cUS) findings until discharge<br>2) At term equivalent age: Brain injury score assessed on brain MRI<br>3) At 24 months: Mental development (Bayley III), incidence of visual, hearing and motor impairment
**Number of Subjects:**	In total, 120 very preterm infants have to be enrolled (n=60 verum, n=60 placebo), to achieve a target sample size of n=100 for secondary outcome MRI at term equivalent age and to achieve a target sample size of n=80 for primary outcome at 5 years of age.
**Diagnosis and Main Inclusion Criteria:**	1. Infants at birth < 32 weeks gestational age and/or < 1'500 g<br>2. IVH grade II-IV (grade IV= haemorrhagic parenchymal infarction)<br>3. Chronological age < 9 days of life (DOL)<br>4. Informed written parental consent
**Main Exclusion Criteria:**	1. Genetically defined syndrome<br>2. Severe congenital malformation adversely affecting life expectancy and/or neurodevelopment<br>3. A priory palliative care<br>4. Unlikely to participate at 5-year follow-up examination
**Study Product, Dose, Route, Regimen:**	Recombinant human EPO, intravenous application, dosage: 2'000 U/kg of body weight, administered three times for loading beginning at DOL 5 (-2/+3 days), which is defined as treatment day 1 (T1), followed by applications 24 hours (-/+2 h) later (T2) and 48 hours (-/+2 h) (T3), and for maintenance at T10 (9 days after T1) and T17 (16 days after T1). Total dosage: 10'000 U/kg.
**Duration of administration:**	5 i.v. applications during three weeks
**Reference therapy, Dose, Route, Regimen:**	Reference is a placebo (saline, 0.9% NaCl).

**Study Schedule:**	Scheduled starting date (First-Subject-In): April 2014<br>Recruitment 4.5 years, approximately until the end of 2018<br>Prospective total duration of study (Last-Subject-Out) December 2023
**Statistical Methodology:**	General linear models will be used to analyse the difference between the two groups. For binary target variables logistic regression models will be applied. Kaplan-Meier curves will be provided for durations of stay.
**GCP Statement:**	This study will be conducted in compliance with the protocol, the current version of the Declaration of Helsinki, and ICH-GCP as well as all national legal and regulatory requirements.
**Definitions:**	IMP dosage is calculated on the basis of body weight. Birth weight has to be used until the actual weight is higher. Then the actual weight has to be used. Infants with more than 1.5 kg receive maximal dosage of 3000 IU.<br>Days of life (DOL) are calculated on basis of calendar days. Hence, calculation always starts with DOL 1 irrespective if DOL 1 has several hours or just few minutes.

STUDY SCHEDULE

Point(s) of time<br><br>**Action**	**In Hospital**					**Follow-up**		
	**Before start of study**	**DOL 1-7**	**DOL 5 (-2/+3) -21**	**DOL 28**	**PMA 36 wks**	**at term equi-valent age**	**2 years of life**	**5 years of life**
Visit 0:<br>- inclusion and exclusion criteria<br>- cranial ultrasound[1]<br>- informed consent	■							
Visit 1: Randomization		■						
Procedure 1: Treatment with IMP[2] five times			■■■■■					
Continuous monitoring[3]		■	■	■	■			
Visit 2: clinical records<br>- Blood count[4]<br>- Cranial ultrasound[1]				■				
Visit 3: clinical records<br>- Blood count[4]<br>- Cranial ultrasound[1]					■			
Visit 4: Brain MRI						■		
Visit 5: BSID-III							■	
Visit 6: K-ABC								■
Study medication			■					
AEs and SAEs			■	■	■[5]			
Blood sampling for biomarkers[6]		■		■	■			

■ compulsory (for medication, adverse events etc.)

1. Cranial ultrasound is performed regularly after birth (DOL 1-7), after 4 weeks (DOL 28) and before discharge at PMA 36 weeks, part of regular controls.
2. IMP: Investigational Medicinal Product
3. Continuous monitoring: heart rate, pulse oximetry, part of regular monitoring
4. Blood counts: haematocrit and haemoglobin, part of regular controls.
5. All new AEs and SAEs that the investigators will be notified of until discharge home have to be reported.
6. Blood sampling is performed only in few study sites as indicated in chapter 6.10

TABLE OF CONTENTS

1 LIST OF ABBREVIATIONS AND DEFINITIONS OF TERMS 7
2 INVESTIGATORS AND STUDY ADMINISTRATIVE STRUCTURE 8
3 ETHICS 11
 3.1 Independent Ethics Committee (IEC) and Regulatory Authorities 11
 3.2 Ethical Conduct of the Study 11
 3.3 Parent Information and Parent Informed Consent 11
 3.4 Amendments 11
4 INTRODUCTION 12
 4.1 Background and Rationale 12
 4.2 Investigational Product 12
 4.3 Preclinical Data 13
 4.4 Clinical Data to Date 13
 4.5 Dose Rationale 14
 4.6 Risk/Benefits and Ethical Considerations 14
5 STUDY OBJECTIVES AND OUTCOMES 16
 5.1 Primary Variable(s) 16
 5.2 Secondary Variables 16
6 STUDY DESIGN AND COURSE OF STUDY 18
 6.1 General Design 18
 6.2 Visit 0 (DOL 1-8, Screening) 18
 6.3 Visit 1 (DOL 1- 8, Randomization) 19
 6.4 Procedure 1 20
 6.5 Visit 2, after completion of study treatment, DOL 28 20
 6.6 Visit 3, postmenstrual age of 36 completed weeks gestation, 36 0/7 22
 6.7 Visit 4, MRI at term equivalent age 23
 6.8 Visit 5, Follow-Up with 2 years (FU2) 25
 6.9 Visit 6, Follow-Up with 5 years (FU5) 26
 6.10 Addendum: Determination of blood and csf biomarkers 28
7 SUBJECT SELECTION AND WITHDRAWAL 29
 7.1 Inclusion Criteria 29
 7.2 Exclusion Criteria 29
 7.3 Subject Recruitment and Screening 29
 7.4 Early Withdrawal of Subjects 29
 7.5 Criteria for Early Withdrawal of Subjects 29
  7.5.1 Data Collection and Follow-up for Withdrawn Subjects 29
 7.6 Early Termination of Study 29
8 TREATMENTS 30
 8.1 Treatments to be Administered 30
 8.2 Identity of Investigational Product(s)Treatment, test drug 30
 8.3 Method of Assigning Subjects to Treatment Groups 30
 8.4 Treatment Compliance 30

8.5 Prior and Concomitant Therapy....30
8.6 Packaging, Labelling and Supply....31
8.7 Blinding....31
8.8 Storage Conditions....32
8.9 Study Drug Accountability....32
8.10 Return or Destruction of Study Drug....32
9 STUDY PROCEDURES....33
9.1 Study-related Procedures....33
9.2 Study-specific Procedures....34
9.3 Sample preparation, storage, and shipment....34
10 SAFETY....35
10.1 Definition of (Serious) Adverse Events....35
10.2 Recording of (Serious) Adverse Events....37
10.3 Assessment of (Serious) Adverse Events....38
10.4 Reporting of Serious Adverse Events and other Safety Related Events....38
10.5 Follow up of (Serious) Adverse Events....39
10.6 Data Safety Monitoring Board....40
10.7 Addendum only for study centres in Switzerland....40
11 DATA QUALITY ASSURANCE....41
11.1 Data evaluation....41
11.2 Routine Monitoring....41
11.3 Inspections....41
11.4 Specification of Source Documents....42
12 STATISTICS....43
12.1 Statistical and Analytical Plans....43
12.2 Endpoints and hypotheses....43
12.3 Planned Analyses....43
12.4 Handling of Missing Data....44
12.5 Determination of Sample Size....44
13 DATA HANDLING AND RECORD KEEPING....46
14 CONFIDENTIALITY....46
15 INSURANCE....47
16 STUDY REGISTRATION....47
17 SIGNATURES....48
REFERENCES....51

1 LIST OF ABBREVIATIONS AND DEFINITIONS OF TERMS

AE	Adverse Event
BW	Birth weight
BPD	Bronchopulmonary dysplasia
CA	Competent Authorities
CEC	Competent Ethics Committee
CRF	Case Report Form
eCRF	Electronic Case Report Form
cUS	Cerebral or cranial ultrasound
DOL	Day of life
DSMB	Data Safety Monitoring Board
EDD	Estimated date of delivery (40 weeks GA)
EPO	Erythropoietin
FU2	Follow up at 2 years
FU5	Follow up at 5 years
GA	Gestational age
GCP	Good Clinical Practice
GMH	Germinal Matrix Haemorrhage
ICH	International Conference on Harmonization
IMP	Investigational Medicinal Product
ISF	Investigator Site File
IVH	Intraventricular Haemorrhage, including GMH and HPI
K-ABC	Kaufman Assessment Battery for Children, German version
MRI	Magnet resonance imaging
NEC	Necrotizing enterocolitis
NICU	Neonatal intensive care unit
PI	Principal Investigator
PHI	Parenchymal Haemorrhagic Infarction
PMA	Postmenstrual age
PVL	Periventricular Leukomalacia
RCT	Randomized Controlled Trial
ROP	Retinopathy of prematurity
SAE	Serious Adverse Event
SD	Standard Deviation
SDV	Source Data Verification
SON-R	Snijders-Oomen non-verbal intelligence test
SOP	Standard Operating Procedure
T	Treatment day
SUSAR	Suspected Unexpected Serious Adverse Reaction
TMF	Trial Master File
WI	Working Instruction

2 INVESTIGATORS AND STUDY ADMINISTRATIVE STRUCTURE

Responsible Staff

● Co-investigator:

Dr. med. Christoph Rüegger, Klinik für Neonatologie, UniversitätsSpital Zürich, 8091 Zürich

Phone +41 44 255 5340, Fax +41 44 255 4442, Email ch.rueegger@gmail.com

● Study Nurse:

Ms Claudia Knöpfli-Lenzin, Klinik für Neonatologie, UniversitätsSpital Zürich, 8091 Zürich

Phone +41 44 255 5342, Fax +41 44 255 4442 Email claudia.knoepfli@usz.ch

● Finances:

Ms Iris Suter, Klinik für Neonatologie, UniversitätsSpital Zürich, 8091 Zürich

Phone +41 44 255 5340, Fax +41 44 255 4442, Email iris.suter@usz.ch

● Pharmacy:

Dr. Karsten Bucher, Kantonsapotheke Zürich, Spöndlistrasse 9, 8006 Zürich

Phone +41 44 255 3211, Fax +41 44 255 4546, Email karsten.bucher@usz.ch

● Biometrician:

Dr. Beate Sick, Abteilung für Biostatistik, Institut für Sozial- und Präventivmedizin, Biostatistik, Universität Zürich, Hirschengraben 84, 8001 Zürich

Phone +41 44 634 4816, Fax +41 44 634 4986, Email beate.sick@ifspm.uzh.ch

● Study Monitoring:

CTC Zürich

● Analysis of MRI data:

PD Dr. med. Cornelia Hagmann, Universitäts-Kinderspital Zürich, Steinwiesstrasse 75, 8032 Zürich

Phone: +41 44 266 71 11, Fax +41 44 266 71 71, Email: cornelia.hagmann@kispi.uzh.ch

● Data Safety Monitoring Board:

Two experts of the Swiss Research Network of Clinical Pediatric Hubs (SwissPedNet) and a Biometrician from the CTU Basel.

1.2. Scientific Advisors

Prof. Dr. Dr. Hannelore Ehrenreich, Full Professor of Neurology and Psychiatry, Division of Clinical Neuroscience
Max-Planck-Institute of Experimental Medicine, Hermann-Rein-Str. 3, 37075 Göttingen, Germany
Phone: +49 551 3899 628, Fax: +49 551 3899 670, Email: ehrenreich@em.mpg.de

Prof. Dr. Max Gassmann, Full Professor and Chairman of Institute of Veterinary Physiology, Vetsuisse Faculty and Zurich Centre for Integrative Human Physiology (ZIHP)
University of Zürich, Winterthurerstrasse 260, CH-8057 Zürich, Switzerland
Phone: +41 44 63 58 803, Fax: +41 44 63 58 932, Email: maxg@access.uzh.ch

1.4. Leading Ethical Committee Responsible for the Primary Investigator

Kantonale Ethikkomission (KEK) Zürich, Stampfenbachstrasse 121, CH-8090 Zürich, Switzerland
Phone: +41 43 259 79 70, Fax: +41 43 259 79 72, Email: info.kek@kek.zh.ch

Participating study centres and local principal investigators

Switzerland:

Aarau	KD Dr. med. Philipp Meyer Schiffer, Chefarzt<br>Abteilung für Neonatologie, Kantonsspital Aarau, Tellstrasse, 5001 Aarau<br>Tel. +41 62 838 4948, Email philipp.meyer@ksa.ch
Basel	Prof. Dr. med. Sven Schulzke, Leitender Arzt<br>Universitäts-Kinderspital beider Basel (UKBB), Spitalstrasse 33, 4056 Basel<br>Tel. +41 61 556 5486, Email: sven.schulzke@ukbb.ch
Bern	PD Dr. med. Mathias Nelle, Leitender Arzt<br>Abteilung für Neonatologie, Inselspital, Bern<br>Tel. +41 31 632 1403, Email: mathias.nelle@insel.ch
Chur	Dr. med. Brigitte Scharrer, Leitende Ärztin<br>Kinderklinik am Kantonsspital Graubünden, Loestrasse 170, 7000 Chur<br>Tel. +41 81 256 6406, Email walter.baer@ksgr.ch
Lausanne	Prof. Dr. med. Anita Truttmann, Leitende Ärztin<br>Service de Néonatologie Clinique Infantile<br>Centre Hospitalier Universitaire, CHUV; 1011 Lausanne<br>Tel. +41 21 314 36 92, Email: anita.truttmann@chuv.ch

St. Gallen Dr. med. Bjarte Rogdo, Oberarzt
Abteilung für Neonatologie, Ostschweizer Kinderspital, Claudiusstrasse 6, 9006 St. Gallen
Tel. +41 71 494 1867, Email Bjarte.Rogdo@kispisg.ch

Zürich Prof. Dr. med Jean-Claude Fauchère
Klinik für Neonatologie, UniversitätsSpital Zürich, Frauenklinikstrasse 10, 8091 Zürich
Tel. +41 44 255 5340, Email jean-claude.fauchere@usz.ch

Germany:

Berlin Prof. Dr. Christoph Bührer, Direktor
Klinik für Neonatologie, Charité Universitätsmedizin Berlin, 13344 Berlin
Tel. +49 30 450 566122, Email: christoph.buehrer@charite.de

Prof. Bührer is the sponsor representative in the EU

Austria:

Wien Ass.-Prof. PD Dr. med. Katrin Klebermaß-Schrehof, Oberärztin
Abteilung für Neonatologie, Pädiatrische Intensivmedizin und Neuropädiatrie
Universitätsklinik für Kinder- und Jugendheilkunde AKH Wien
Währinger Gürtel 18-20; 1090 Wien
Tel. +43 1 40400 32320, Email: katrin.klebermass-schrehof@meduniwien.ac.at

3 Ethics

3.1 Independent Ethics Committee (IEC) and Regulatory Authorities

Before this study will be conducted, the protocol, the proposed subject information and consent form as well as other study-specific documents will be submitted to a properly constituted Independent Ethics Committee (IEC) and regulatory authorities (Swissmedic) in agreement with local legal requirements, for formal approval. Any amendment to the protocol must be approved by these institutions.

The decision of the IEC and Swissmedic concerning the conduct of the study will be made in writing to the Sponsor, the Principal Investigator, and all local PIs before commencement of this study.

3.2 Ethical Conduct of the Study

The study will be carried out in accordance with principles enunciated in the current version of the Declaration of Helsinki, the guidelines of Good Clinical Practice (GCP) issued by ICH, and Swiss regulatory authority’s requirements.

IEC and regulatory authority will receive annual safety and interim reports and be informed about study stop/ end in agreement with local requirements.

3.3 Parent Information and Parent Informed Consent

The investigator must explain to parents of each subject the nature of the study, its purpose, the procedures involved, the expected duration, the potential risks and benefits and any discomfort it may entail. Parents of each subject must be informed that the participation in the study is voluntary and that they may withdraw from the study at any time and that withdrawal of consent will not affect subsequent medical treatment of theirs infant.

The parents must be informed that the medical records of theirs infant may be examined by authorized individuals other than theirs treating physician.

All parents of subjects for this study will be provided a parent information sheet and a consent form describing this study and providing sufficient information for parents to make an informed decision about their participation in this study.

The parent information sheet and the consent form will be submitted with the protocol for review and approval for the study by the IEC. The formal consent of parents, using the approved consent form, must be obtained before theirs infant is submitted to any study procedure.

The parents should read and consider the statement before signing and dating the informed consent form, and should be given a copy of the signed document. The consent form must also be signed and dated by the investigator (or his designee) and it will be retained as part of the study records.

3.4 Amendments

Substantial amendments are only implemented after approval of the CEC and CA respectively.

Under emergency circumstances, deviations from the protocol to protect the rights, safety and well-being of human subjects may proceed without prior approval of the sponsor and the CEC/CA. Such deviations shall be documented and reported to the sponsor and the CEC/CA as soon as possible.

All Non-substantial amendments are communicated to the CA as soon as possible if applicable and to the CEC within the Annual Safety Report (ASR).

4 INTRODUCTION

This document is a protocol for a human research study.

4.1 Background and Rationale

Approximately 1% of all infants are born very preterm with a birth weight (BW) <1’500 g and a gestational age (GA) below 32 completed weeks of gestation (VLBW: very low birth weight). Although more than 85% of these VLBW infants survive, roughly 40% of survivors are affected by neurodevelopmental impairment and learning disabilities upon entering school.[1, 2] One of the most consistent predictors of neurodevelopmental impairment is intraventricular haemorrhage (IVH),[3-6] which occurs in approximately 20% of all VLBW infants, mostly within the first few days after birth. Among VLBW infants, increased prematurity at birth, higher rates of IVH and a higher likelihood of neurodevelopmental impairment are directly linearly related.[2] Infants with large IVH (grade III or IV, the latter of which is now often also termed “haemorrhagic parenchymal infarction”[7]), especially those requiring shunt insertion for posthaemorrhagic hydrocephalus, are the most severely affected,[3, 8] though infants with low-grade IVH (grade I or II) have reduced brain cortical volume[9] and lower developmental scores.[10] In addition to immediate parenchymal damage and free iron toxicity, IVH results in the loss of germinal matrix precursor cells and negatively affects contralateral cerebellar growth,[11, 12] which contributes to long-term cognitive, learning, and behavioural disability.[13] There are very few evidence-based measures to reduce the risk of IVH,[14] such as foetal lung maturation or late cord clamping. The incidence of IVH among VLBW infants has declined only slightly over the last two decades, in contrast to other forms of brain damage such as periventricular leukomalacia (PVL).[3, 15-17] Except for shunt insertion to divert cerebrospinal fluid in infants with posthaemorrhagic hydrocephalus and possibly the removal of blood clots in infants with high-grade IVH,[18] there is no treatment for established IVH, and no medical therapies exist to ameliorate the neurodevelopmental sequelae of IVH.

The second important predictor of neurodevelopmental impairment in VLBW infants is cerebral white matter injury which may follow IVH but occurs also independently. Cerebral white matter injury includes diffuse PVL and cystic PVL (cPVL) which are frequently accompanied by neuronal/axonal disease, affecting the cerebral white matter, thalamus, basal ganglia, cerebral cortex, brain stem, and cerebellum. Recently, this constellation of PVL and neuronal/axonal disease has been termed "encephalopathy of prematurity".[19] As the term white matter injury is much more common we will keep it here. Cranial ultrasound can detect especially cPVL but fails to discriminate the more subtle manifestations of white matter injury[1, 2,20]. Whereas IVH presents as an acute event in VLBW infants within the first week of life, the diagnosis of white matter injury is usually made between 2 and 5 weeks of life.[21,20].

Together, as there are no medical therapies to ameliorate the neurodevelopmental sequelae of IVH and of cerebral white matter injury which may follow IVH but occurs also independently, the rationale for this study is to test recombinant human erythropoietin (EPO). It has been shown to be protective against hypoxic-ischaemic and inflammatory injuries in a broad range of tissues and organs. EPO has been shown to have neuroprotective and neurotrophic activity in animals after acute brain damage as well as in adult stroke patients.

4.2 Investigational Product

There are many companies manufacturing recombinant EPO. In the prospective multi-centre randomised placebo-controlled trial (RCT) on prophylactic EPO in VLBW infants in Switzerland (‘Does Erythropoietin improve outcome in very preterm infants’, StV-36/04; www.clinicaltrials.gov; NCT00413946) [22] which closed for recruitment in March 2012 as the required number of 420 VLBW patients was achieved, we used Recormon with good results. Hence, Recormon will also be used in the here described new trial, EpoRepair. Recormon is manufactured by Roche Pharma (Reinach, Schweiz) and contains Epoetinum beta, genetically engineered EPO in CHO-cells.

4.3 Preclinical Data

Apart from stimulating production of red blood cells in the bone marrow, EPO has been shown to exert neuroprotective action in a variety of animal models.[23-28]

Studies in rats and sheep have shown greater clearances, shorter half-lives, shorter residence times and greater distribution volumes after an intravenous bolus of EPO in foetuses and newborns compared to adults.[29] Consequently, the recommended dose per kg of body weight for prevention of anaemia is higher in preterm infants than in adults.

To be neuroprotective, EPO must cross the blood-brain barrier. This has been shown to occur in rats after experimental brain injury[30] and in sheep and nonhuman primates.[31]

4.4 Clinical Data to Date

EPO administration has been found to be beneficial and safe in RCTs involving adult patients with ischemic stroke[32] or schizophrenia,[33, 34] and in term new-born infants with perinatal asphyxia.[35, 36]

In RCTs involving preterm infants, EPO has been shown to reduce the need for red blood cell transfusions in preterm infants[37-39] and is one of the few drugs specifically licensed for use in preterm infants at least in Germany and USA. For the purpose of stimulating erythropoiesis in preterm infants, 250-300 IU/kg of EPO are administered 3-5 times a week intravenously or subcutaneously.

Observational data from VLBW infants treated with EPO mainly to prevent red blood cell transfusions point to a role of EPO in neuroprotection and repair. First, EPO serum concentrations > 500 mU/ml in VLBW infants have been found to be associated with improved neurodevelopmental scores at 18-24 months of age.[40] Second, greater cumulative doses of EPO during the first six weeks of life were associated with higher adjusted neurodevelopmental scores.[41] Third, a recent retrospective analysis strongly suggests that EPO improves long-term neurodevelopmental outcomes in VLBW infants with intraventricular haemorrhage (IVH), without apparent effects in VLBW infants without IVH.[42] A significantly higher percentage of VLBW infants with IVH who received EPO subsequently displayed normal IQ at school age, compared to VLBW infants with IVH who did not receive EPO (infants with normal IQ 6-7 years after EPO was 49% vs. 31% without EPO). As most infants received EPO within days or weeks after experiencing an IVH, the proposed mode of action of EPO is thought to be the facilitation of repair and the sustainment of neuronal growth and differentiation after brain injury, rather than protection against damage.

Juul et al. showed a large increase of endogenous EPO in the cerebrospinal fluid (CSF) of human newborns who experienced asphyxia (225±155 mU/ml, range 7 to 2'350 mU/ml) compared to control infants (6±1 mU/ml).[43] The minimal effective dose for neuroprotection in preterm infants is not known. In an adult stroke trial, 100'000 IU of EPO was divided into 3 doses and given intravenously and was shown to be safe and effective.[44] In rats, 5'000 IU/kg had the same neuroprotective effect as 10'000 IU/kg; both were well tolerated.[45]

Two pilot trials conducted in preterm infants with the primary outcome of neuroprotection have found doses of EPO that have been well tolerated; one trial administered 3'000 U/kg of EPO intravenously at 3, 12-18 and 36-42 h after birth,[22] and the other administered 500, 1'000 or 2'500 U/kg of EPO 3 times at 24-h intervals.[46,47] At a dose of 1'000 U/kg or more, the peak serum EPO concentrations were in the range that is neuroprotective in experimental animals.[31] Single-dose pharmacokinetics of EPO has been studied in preterm infants after intravenous and subcutaneous administration.[48,49] After a dose of 200 U/kg intravenously, the mean (±SD) volume of distribution was 300 (±72) ml/kg, total body clearance was 27 (±9) ml/h*kg, the half-life was 8 (±3) hours and the maximum serum concentration was 711 (±166) U/l. In the stroke trial.[44], serum levels rose to 5649 (±903) mU/ml after the third intravenous dose of 33'000 IU of EPO. This resulted in CSF concentrations of 17.1 (±5.6) mU/ml, which is 60 to 100 times that of non-treated patients.

The primary endpoint of the prophylactic EPO RCT in VLBW infants (StV-36/04; www.clinicaltrials.gov; NCT00413946) is neurodevelopmental assessment at 2 years of life, including the Bayley Scales III of Infant Development and the determination of presence or absence of impaired motor function (cerebral palsy) and neurosensory function (blindness or deafness)[22]. As recruitment was completed in March 2012 assessment of the last infant will be in 2014. However, one secondary endpoint is blinded neuro-imaging of 100 VLBW infants by means of advanced MRI at term equivalent age. A significantly lower white matter injury score (6.67±0.16 vs. 7.3±0.29, p=0.004), with fewer white matter abnormalities and less periventricular white matter loss on conventional MRI, was found in the EPO-treated group even after adjustment for GA and BW. No significant differences were found in grey matter and cerebellum injury score or in measures of cerebellar growth and maturation.[50]

In adults, EPO treatment has been associated with some adverse events, including hypertension, thrombotic events, seizures, and polycythaemia. In VLBW infants, long-term EPO treatment has not been associated with any of these complications.[51, 52] Furthermore, Fauchere et al.[22] did not identify significant adverse effects of an early high-dose EPO treatment in very preterm infants and also the second study on high dose EPO in VLBW infants concluded that EPO is safe [47]. In a Cochrane meta-analysis of early treatment studies, Ohlsson and Aher[37] found an increased frequency of severe retinopathy of prematurity. However, more recent data could not confirm this adverse effect in VLBW infants [53] or in rodents treated with high-dose EPO.[54].

4.5 Dose Rationale

As argued in the section above, we consider a dose of 2'000 IU/kg given 3 times over 3 subsequent days to be sufficient to achieve a CSF concentration of 5 U/ml, assuming a serum to CSF ratio of 1 to 200 (loading, cumulative dose 6'000 IU/kg). Two following administrations at one week and at two weeks after loading are planned in terms of maintaining increased CSF concentrations during repair after the first weeks after the insult (maintenance, cumulative dose 4'000 IU/kg).

4.6 Risk/Benefits and Ethical Considerations

Approximately 40% of the overall burden of intellectual disability of VLBW preterm infants is attributable to IVH.[3] There is presently no established medical therapy to ameliorate the neurological sequelae of IVH in VLBW infants.[14] Observational data suggest that recombinant human EPO administered to VLBW infants with IVH substantially improves long-term cognitive outcomes[40-42] and increases the proportion of school-age children with a normal IQ from 24% to 62%, which is similar to VLBW infants without IVH.[42] These data, however, are observational and therefore do not allow for any firm conclusions or recommendations. The hypothesis generated by these data calls for confirmation or refutation by an RCT designed to address this question.

Recombinant human EPO administered to VLBW infants for both prolonged periods of time[37,39,38] and at high doses[2247] has been found to be remarkably safe. Concerns about the possibility of an increased rate of retinopathy of prematurity with very early administration of EPO[37] have not been confirmed,[42, 53].

In addition and most important, the safety data of the recently completed first high-dose EPO study [22] are very encouraging and will be presented on the annual meeting of the European Society for Paediatric Research (ESPR) 2012 in Istanbul. The major finding is: No significant adverse effects of early high-dose rhEpo treatment in very preterm infants were identified and the detailed data are given here:

	Epo	Placebo	p
N	206	189	
Gestational age: weeks, mean (SD)	29.3 (1.6)	29.3 (1.6)	0.99
Birth weight: g, mean (SD)	1232 (373)	1231 (314)	0.99
Girls: n (%)	85 (41%)	73 (39%)	0.67
Haematocrit day 7-10: %, mean (SD)	47.3 (7.9)	44.8 (7.1)	**0.002**
Death: n (%)	12 (5.9%)	11 (5.9%)	0.99
Sepsis: n (%)	24 (10.9%)	25 (13.2%)	0.75
Bronchopulmonary dysplasia: n (%)	21 (10.8%)	24 (13.5%)	0.53
Retinopathy of prematurity: n (%)	12 (6.4%)	14 (8.3%)	0.64
Intraventricular haemorrhage: n (%)	38 (18.4%)	28 (14.8%)	0.41
Haemangioma: n infants (%)	35 (17.0%)	34 (18.0%)	0.89
Severe adverse events	46	45	0.82
Days in hospital: mean (SD)	54.2 (25.1)	55.3 (25.1)	0.67

Human recombinant EPO specifically licensed for the use in humans (Epoietin-beta), and not biosimilar products will be administered.[55]

As this trial offers a unique chance to receive a potentially beneficial treatment for IVH, the majority of parents of potential subjects are expected to be interested in enrolment in the trial. In the European Multicentre Erythropoietin Beta Study that called for randomisation at 3 d of age, 73% of parents of VLBW infants agreed to participate.[56] A high rate of participation at 5-year follow-up examinations will require consistent contact with the enrolled families. Follow-up rates were 87% in the single-centre analysis of improved neurodevelopmental outcomes following EPO administration in VBLW infants[42] and 78% in the population-based EPICure study.[1]

Given that white matter injury is the second most frequent predictor of neurodevelopmental impairment in VLBW infants following IVH and taking into account that EPO treatment significantly lowered white matter injury scores on MRI,[50] it may be concluded that VLBW infants with profound white matter injury should also be included in this EpoRepair study. While this is true in principle, white matter injury is typically diagnosed between 2 and 5 weeks after birth, by which time enteral feeding is often already established and no intravenous line is available for administration of the study drugs. Moreover, when white matter injury becomes visible by ultrasound, especially cPVL, white matter injury has already progressed to a later stage of disease.[21,20] Thus, we will limit our study to VLBW infants with an early brain injury.

5 STUDY OBJECTIVES AND OUTCOMES

The purpose of this study is to evaluate the effect of intravenously administered EPO as compared to placebo in VLBW infants with IVH (grade II-IV) diagnosed by cranial ultrasound at a chronological age < 8 d on neurological development. There is presently no established medical therapy to ameliorate the neurological sequelae of IVH in VLBW infants.[14]

Primary Outcome: To assess the efficacy of EPO on decreasing neurological sequelae of IVH in VLBW infants intelligence quotient will be assessed at 5 years of age.

5.1 Primary Variable(s)

Intelligence quotient assessed by the Kaufman Assessment Battery for Children (Kaufman-ABC) (German version) [57] at 5 years of age will be the primary efficacy outcome variable.[42]. Children with speaking problems at 5 years will be assessed by an alternative non-verbal intelligence test, the Snijders-Oomen non-verbal intelligence test (SON-R 2.5-7, German version). [58]

Neurodevelopmental follow-up examinations of VLBW infants at 18-24 months using the Bayley Scales III of Infant Development (BSID-III) will be an intermediate variable. However, as 2 year outcome assessment is limited in predicting later cognitive functions,[1, 59, 60] the 5 year outcome will be the main outcome variable.

The vast majority of children will remain on their 5-year IQ trajectory, and there is good agreement between classification of cognitive abilities at 5 and 11 years of age.[61] In the retrospective analyses on neurodevelopmental outcome of VLBW infants after EPO, differences between EPO-treated infants and controls were at the borderline of significance at 2 years of age[40, 41] whereas cognitive scores diverged between EPO-treated children and controls at 3-4 years and did more so at 6-7 years of age.[42]

Children will be classified as either being normal or having minor or major impairment according to the overall results of the tests as summarized in the table below. Children with intelligence incapable of measurement because of impairment severity will be assigned IQ scores below the lowest standard score of Kaufman-ABC and the SON-R 2.5-7.

Normal development: All of the following:
- Normal neurological evaluation, IQ > 84
- No neurodevelopmental deficits

Minor impairment: One or more of the following problems:
- Subnormal cognitive abilities (IQ, 70-84)
- Gross and fine motor activity deficits
- Disorders of language development
- Visual and auditory deficiencies
- Attention disorders
- Abnormal socioemotional development

Major impairment: One or more of the following problems:
- Cerebral palsy
- Intellectual disability (US mental retardation) with IQ < 70
- Blindness, deafness
- Intractable epilepsy

5.2 Secondary Variables

1) The occurrence of mortality, white matter disease (periventricular leucomalacia), septicaemia, necrotising enterocolitis, bronchopulmonary dysplasia (oxygen dependency at 36 weeks postmenstrual age), retinopathy and length of stay in hospital will be documented in each infant.

2) Cranial ultrasound is a useful point of care method to detect, confirm and monitor brain damage including IVH. However, cranial ultrasound lacks to detect diffuse white matter injury. Brain MRI is able to detect more subtle injury and brain growth. Furthermore, quantitative MR techniques allow to describe volumetric and microstructural changes. As EPO is hypothesized to act by affecting neuronal repair, growth and differentiation, MRI at term equivalent age might help to establish early markers and identify structural patterns of IVH-mediated brain damage and EPO-aided repair.
3) Outcome measures of MRI examination at term equivalent age, including brain maturation score and white matter and grey matter injury scores.
4) At 24 months of age corrected for prematurity Bayley Scales of Infant Development (BSID-III) and the presence or absence of impairment of motor function (cerebral palsy) and neurosensory function (blindness or deafness) will be assessed.

This study will use established definitions for tests accepted and introduced within the Swiss Neonatal Network & follow-up group.

6 STUDY DESIGN AND COURSE OF STUDY

6.1 General Design

This is a multicentre, placebo-controlled, prospective, randomised, double-blind (verum:placebo = 1:1) clinical study. The effect of EPO treatment on decreasing neurological sequelae of IVH (grade II-IV) will be studied in 100 VLBW infants. All VLBW infants are routinely screened for IVH in the first days after birth. More than 96% of all cases of IVH occur within the first 5 days of life IVH. If subjects fulfil inclusion criteria and parental written informed consent is given at a chronological age < 9 day of life, subjects will be enrolled in the study.

DOL is calculated on basis of calendar days. Hence, calculation always starts with DOL 1 irrespective if DOL 1 has several hours or just few minutes.

Flowchart, day of life (DOL) and treatment day (T)

Visit 0 (**DOL 1- 8**)	Obtaining parental approval (informed written consent)<br>for eligible infants:<br>- GA at birth < 32 weeks and/or BW < 1'500 g<br>- IVH grade II-IV at chronological age < 9 d as assessed by cUS
Visit 1 (**DOL 1- 8**)	Randomization in EPO vs. placebo group
Procedure 1 (DOL 5 -2/+3 days) start of study treatment	T1: 2'000 IU EPO/kg intravenously vs. placebo<br>T2 (24 h ± 2 h after T1): 2'000 IU EPO/kg intravenously vs. placebo<br>T3 (48 h ± 2 h after T1): 2'000 IU EPO/kg intravenously vs. placebo<br>- Three doses are considered for loading<br>T10 (9 days after T1): 2'000 IU EPO/kg intravenously vs. placebo<br>T17 (16 days after T1): 2'000 IU EPO/kg intravenously vs. placebo<br>- Two doses are considered for maintenance therapy
Visit 2 (DOL 28)	cUS and blood count, both are parts of standard care, recording of relevant clinical data as given below and on eCRF 'visit 2' (± 3 days)
Visit 3 (PMA 36 wk)	cUS and blood count, both are parts of standard care, recording of relevant clinical data as given on eCRF 'visit 3' (± 3 days)
Visit 4	MRI at term equivalent age (40 0/7 ± 3 weeks postmenstrual age)
Visit 5	18-24 months of life, Bayley Scales III of Infant Development (BSID-III) is mandatory in Switzerland and Germany
Visit 6	5 years of life, Intelligence quotient (K-ABC) at 5 years of age will be the primary efficacy outcome variable

The study is scheduled to begin on April 2014. The in-hospital phase of the study will last until the infant is mature enough for safe discharge home, approximately at term equivalent age. All patients will be followed up for developmental outcome at 18-24 months after term and at five years. Therefore, the whole study will last five years for each participating child.

6.2 Visit 0 (DOL 1-8, Screening)

Only VLBW infants are eligible with IVH grade II-IV, for which all VLBWs are routinely screened by cUS. If IVH grade II-IV is present and VLBW infants are eligible according to inclusion and exclusion criteria listed below, parents will be asked for participation.

Inclusion criteria

The trial will include female and male preterm infants with the following criteria:

- Informed written parental consent
- IVH (grade II-IV)

- GA < 32 0/7 weeks and/or BW < 1'500 g at birth. Comment: There is a steep rise in the incidence of IVH with decreasing GA and decreasing BW
- Chronological age < 9 d. Comment: More than 96% of all cases of IVH happen within the first 5 d of life. In contrast, IVH in term infants is a rare event and follows a rather different clinical course.

Exclusion criteria
Infants with the following characteristics will be excluded:

- Genetically defined syndrome
- Severe congenital malformation adversely affecting life expectancy or neurodevelopment
- Admitted a priori for palliative care
- Unlikely to participate at 5-year follow-up examination

At this time point the following items will be recorded, also given on eCRF visit 0:

- Date of visit
- Date and time of birth
- Location of birth, if outborn specify the place
- GA (weeks and days) at birth and BW in g
- IVH maximal extend

For cUS the following coronal and sagittal sections are recommended to be performed:

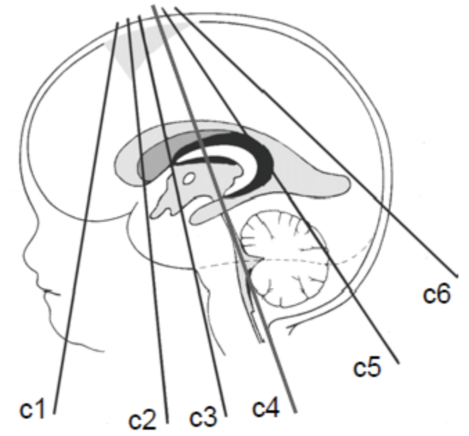

Six coronal sections

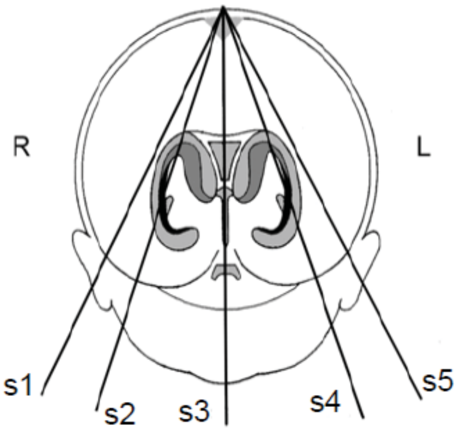

Five sagital sections

6.3 Visit 1 (DOL 1- 8, Randomization)

After checking for inclusion and exclusion criteria and obtaining parental written informed consent before DOL 5 -2/+3 days, each enrolled subject will receive a study number and subsequently the predefined study drug, either verum or placebo. Lists with study numbers and randomly assigned study drugs will be designed by the CTC Zürich in cooperation with the study biometrician, blinded to the study sponsor, all investigators and all involved staff. The study centres will be randomised separately and the local pharmacies will host the respective lists.

6.4 Procedure 1

Study treatment is described above, see 6.1. After treatment unused investigational drug and empty packages will be stored at the local investigational sites until the study monitor will have controlled and released the unused investigational drug and empty packages. Then they will be disposed.

IMP dosage is calculated on the basis of body weight. Birth weight has to be used until the actual weight is higher. Then the actual weight has to be used. Infants with more than 1,5 kg receive maximal dosage of 3000 IU.

6.5 Visit 2, after completion of study treatment, DOL 28

Recording of adverse events (AEs) will be done from first study treatment DOL 5 (-2/+3 days) until discharge home from hospital. Specific AEs will be recorded without time limit and include ROP grade >1 or Plus diseases, BPD if classified as severe at 36 PMA, and anaemia. For further details see chapter 10.
At this time point of DOL 28, when study treatment is completed, course of medication will be recorded finally, including all deviations. In addition, few maternal antepartal characteristics will be recorded which are known to affect morbidity of infants. Infants characteristics well also recorded and the following items will be recorded, also given on eCRF visit 1:

- Date of visit
- Nationality of child
- Language(s) at home
- Number of infants in this birth, if multiple birth indicate the number of this infant
- Mothers age in years at delivery
- Gravida
- Para
- Perinatal steroids, unknown, incomplete or complete
- Prolonged premature rupture of the membranes (PPROM), present or absent (PPROM is defined as rupture of membranes more than 18 hours before delivery and before 37 weeks of gestation.)
- Chorioamnionitis clinical just prior to delivery, present or absent (Clinical chorioamnionitis is diagnosed in women who had three or more of the following signs: leukocytes >20,000/µl, C-reactive protein (CRP) >40 mg/dl, temperature >38°C, maternal tachycardia >100 bpm, fetal tachycardia >160 bpm.)
- EPH-Gestosis (Preeclampsia, PE), present or absent (PE is defined as hypertension (≥140 mm Hg systolic and ≥90 mm Hg diastolic blood pressure) and significant proteinuria ≥300 mg/24h, or ≥ ++ in urine dipsticks.)
- Mode of delivery, indicate if vaginal spontaneous, vaginal instrumental, caesarean section elective or secondary
- Umbilical artery pH
- Apgar score at 5, and 10 minutes
- Crib score, including lowest FiO2, highest FiO2, and worst base deficit, all before 12 hours of life
- Weight (g) and SD from mean weight at birth
- Head circumference (cm) and SD from mean length at birth
- Length (cm), SD from mean length at birth
- First Hct in % and first Hb in g/dl on admission at NICU
- EPO medication:
  - First EPO dose in ml, date and time of application, (T1)
  - Second EPO dose in ml, date and time of application, (T2)
  - Third EPO dose in ml, date and time of application, (T3)

- Fourth EPO dose in ml, date and time of application, (T10)
- Fifth EPO dose in ml, date and time of application, (T17)

- Weight (g) and SD from mean weight at DOL 28
- Head circumference (cm) and SD from mean length at DOL 28
- Length (cm), SD from mean length at DOL 28
- Hct in % and Hb in g/dl at DOL 28
- Complete cUS findings at DOL 1-4 and DOL 28
-

cUS at DOL 28, graded in normal, moderately abnormal and severely abnormal brain injury

Comment: Findings of cUS will be graded in normal, moderately abnormal and severely abnormal brain injury, see below. This grading is a standard procedure in all neonatology units with small variations. cUS is a secondary outcome measure and performed in visit 0, visit 2, and visit 3. To unify grading it is defined below. In addition, correct grading will be supervised by the involved study staff.

Definition of normal, moderately abnormal and severely abnormal brain injury by cUS is as follows:

- Normal brain
    1. No cysts
    2. No ventricular dilatation
    3. No thinning of corpus callosum
    4. No enlargement of extracerebral spaces
    5. Normal cortical grey matter
- Moderately abnormal brain
    1. PVL grade 1 with either homogeneous or inhomogenous increased echogenicity
    2. Any IVH, including GMH and PHI
    3. Ventricle dilatation < 97 centile according to Levene
- Severe brain injury will be defined as:
    1. IVH with ventricular dilatation
    2. Posthaemorrhagic ventricular dilatation > 97 centile
    3. Parenchymal haemorrhagic infarction (PHI)
    4. Cystic PVL
    5. Cerebral atrophy
    6. Cerebellar haemorrhage

Definition of ventricular dilatation, including a normogram for the 97th centile and 4 mm above 97th centile: The ventricular index is the distance between the lateral wall of the body of the lateral ventricle to the falx in the coronal plane just posterior to the foramen of Monro. (Levene, Arch Dis Childh 1981;56:900-904)

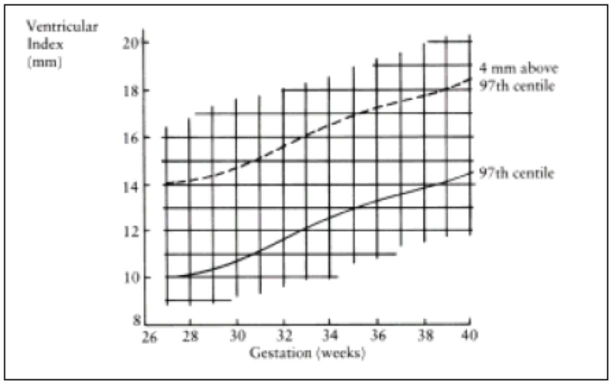

Course of haemorrhage until DOL 28:

Posthaemorrhagic hydrocephalus, graded 0-3:

- 0) Restitution ad integrum – no remaining lesions, date of last follow up
- 1) Persisting posthaemorrhagic hydrocephalus without any drainage
- 2) Persisting posthaemorrhagic hydrocephalus with repetitive but transient csf-drainage, indicate if LP, or Rickham, or both and indicate for how long
- 3) Posthaemorrhagic hydrocephalus with permanent csf-drainage, e.g. VP-Shunt

Convulsions:

- If present and/or any antiepileptic therapy, if yes specify treatment including duration

Other abnormalities or medications related to haemorrhage

- if yes specify

6.6 Visit 3, postmenstrual age of 36 completed weeks gestation, 36 0/7

At this time point the following items will be recorded, also given on SecuTrial form Visit 3. This includes items first recorded at GA 36 0/7 and a summary of treatments and complications of whole hospitalisation. If discharge home is later, some items have to be completed immediately after discharge:

- Date of visit
- Weight (g) and SD from mean weight at GA 36 0/7
- Head circumference (cm) and SD from mean length at GA 36 0/7
- Length (cm), SD from mean length at GA 36 0/7
- Hct in % and first Hb in g/dl at GA 36 0/7

cUS at GA 36 0/7, graded in normal, moderately abnormal and severely abnormal brain injury according to definitions given in visit 2

Course of haemorrhage until visit 3:

Posthaemorrhagic hydrocephalus, graded 0-3:

- 0) Restitutio ad integrum – no remaining lesions, date of last follow up
- 1) Persisting posthaemorrhagic hydrocephalus without any drainage
- 2) Persisting posthaemorrhagic hydrocephalus with repetitive but transient csf-drainage, indicate if LP, or Rickham, or both and indicate for how long
- 3) Posthaemorrhagic hydrocephalus with permanent csf-drainage, e.g. VP-Shunt

Convulsions:

- If present and/or any antiepileptic therapy, if yes specify treatment including duration

Other abnormalities or medications related to haemorrhage

- if yes specify

Other clinical parameters until visit 3:

- Respiratory support at DOL 28 to define if mild BPD is present; indicate if $FiO_2 > 0.21$ and/or any form of positive pressure support, CPAP or mechanical ventilation
- Respiratory support at 36 0/7 to define if moderate or severe BPD is present. BPD moderate if $FiO_2 > 0.21$ but <0.3; BPD severe if CPAP or mechanical ventilation and/or $FiO_2 \geq 0.3$. These definitions are in accordance with those of the NIH adapted by the Swiss society of neonatology.
- Supplemental O_2
  - Last day of supplemental O_2, give date
  - Total days of supplemental O_2
  - Total days of mechanical ventilation
  - Total days of CPAP
- Surfactant treatment
- Treatment with postnatal steroids, given or not given
- PDA prophylaxis, if yes indicate if indomethacin or ibuprofen
- PDA treatment, if yes indicate if indomethacin, ibuprofen, or surgical ligation
- Pneumothorax, if yes, localization
- NEC, date of onset
- FIP, date of onset
- Sepsis, date of onset, indicate if blood culture positive

- Meningitis, date of onset, indicate if csf culture positive
- Surgery
  - none
  - minor, specify if herniotomy, vp-shunt implantation, DA-ligation
  - major, specify
- Haemangioma
- ROP, if yes indicate maximum grade and if Plus disease
  - intervention, if yes specify if laser therapy or other
- Other secondary diagnoses
- Blood transfusion record, only EC-transfusion, if yes indicate amount
- Death
  - Date of death
  - Cause of death
  - Autopsy, if yes summarize the main findings

Recordings at discharge
- Date and GA at first discharge home
- Weight (g) and SD from mean weight at discharge
- Head circumference (cm) and SD from mean length at discharge
- Length (cm), SD from mean length at discharge
- Feeding at discharge, specify if breast feeding/bottle drinking or gastric tubing
- Medications at discharge, including anticonvulsants and diuretics, specify
- Home monitoring
- Home oxygen

Recordings of transfer during hospitalization
- Transfer before discharge at home
- Date of transfer
- Temporal relation of the transfer to the study treatment, during medication/after
- Name of hospital
- Temporary or permanent transfer
- Reason for transfer

6.7 Visit 4, MRI at term equivalent age

At term equivalent age, which is 40 0/7 ± ~~2~~ 3 weeks PMA, cranial MRI will be performed and brain damage will be recorded by a scoring system as given below. Few additional items will be recorded. All this is given on SecuTrial form Visit 4.

- Date of MRI examination and place of MRI examination

Anthropometrics
- Weight (g), SD from mean weight.
- Head circumference (cm), SD from mean circumference.

Imaging:
- MRI system used
- Sequences (must be performed as suggested) T1, T2, Diffusion Tensor Imaging (DTI), H spectroscopy
- Sequences will be performed in the following order:
  - T2w ax.
  - t2_spc_sag_p2_iso_0.9
  - T1_mprage_sag
  - ep2d_dti_30dir_b0_650
  - t2_swi3d_tra_1.25mm

- ASL_3D_tra_iso
- ep2d_bold_p2_3mm_rest
- t1_mp2rage_sag_p3_iso_Lsne
- svs_se_30_wm
- svs_se_30_gm

Brain maturation score:
Scoring of the characteristics brain maturation (M1-M7), cortical infolding (C1-C6), germinal matrix (G1-G4), and bands of migrating glial cells (Gl1-Gl4) will be performed according to the definitions given below. Each characteristic is sub-specified by various items, for each item present = 1, absent = 0. The resulting sum provides the score of each characteristic and the total sum will provide the total maturation score.
Myelination Score (M1-M7):

- M1 Myelination evident in brainstem, cerebellar peduncle, inferior colliculus, cerebella vermis
- M2 Subthalamic nuclei, globus pallidus, ventrolateral thalamus
- M3 Caudal portion of the posterior limb of the internal capsule (PLIC)
- M4 Complete PLIC
- M5 Optic radiation
- M6 Corona radiata
- M7 Anterior limb

Cortical infolding Score (C1 -C6):

- C1 Frontal and occipital cortex completely smooth
- C2 Frontal cortex still smooth, some sulci evident
- C3 Frontal and occipital cortex similar number of convolutions
- C4 Frontal and occipital cortex folded and rich in sulci
- C5 Frontal and occipital WM separated into strands by deeper sulci
- C6 as C5 but WM now isotence with gray matter on T1-weighted images

Germinal Matrix Score (G1 -G4):

- G1 Matrix seen in posterior horn, at caudothalamic notch (CTN) and anterior horns of
- lateral ventricles
- G2 Matrix evident at CTN and anterior horns only
- G3 Matrix at anterior horns alone
- G4 No matrix evident

Bands of migrating glial cells Score (Gl1-Gl4)

- Gl1 Broad bands with additional narrower bands
- Gl2 Broad band alone
- Gl3 Narrow band alone
- Gl4 No bands seen

White matter and grey matter injury score
The scores for white matter injury (WM1-WM5, according to Woodward et al. NEJM, 2006) and gray matter injury (GM1-GM3) are more complex. They are subspecified by various items, whereas normal = 1, mild abnormal = 2, and moderate to severe abnormal = 3. This is given in detail below.
White matter injury score (WM1-WM5)

- WM1 White matter signal abnormality: T1 and T2 weighted signal abnormalities in the WM best observed in the axial imaging.
  1 = normal
  2 = mild abnormality
  3 = moderate- severe abnormality
- WM2 Periventricular WM loos: T1 and T2 weighted analysis of the ratio of periventricular WM to ventricular volume in both axial and sagittal imaging.
  1 = normal

2 = mild abnormality
3 = moderate- severe abnormality

- WM3 Cystic abnormalities:
  1 = no cysts
  2 = less than 2mm single focal cyst
  3 = multiple cysts or single larger cyst > 2mm
- WM4 Ventricular dilatation: based on combined T1-T2 weighted imaging in both axial and sagittal imaging:
  1 = normal
  2 = mild dilatation resulting in mild rounding of the frontal
  3 = moderate -severe enlargement of the frontal, temporal
- WM5 Thinning of corpus callosum: based on the T1 and T2 weighted imaging:
  1 = normal with thick corpus callosum visible in all views
  2 = focal thinning in the corpus callosum often visible in the mid . . .
  3 = global thinning across the entire corpus callosum

Gray matter injury score (GM1-GM3):

- GM1 Presence of gray cortical signal abnormality: high signal intensity in the cortex on axial T1 and/or loss of cortical ribbon signal on axial T2-weighted MRI:
  1 = normal
  2 = mild abnormality
  3 = moderate- severe abnormality
- GM2 Quality of gyral maturation (MRI):
  1 = normal for 40 weeks
  2 = to 4 weeks delay
  3 = more than 4 weeks delay
- GM3 Size of subarachnoidal space: Score based on the sagittal T1 and coronal T1 or T2-weighted. MR images for the size of the subarachnoid space in relation to the cerebral mantle.
  1 = Small subarachnoid space barely visible
  2 = mildly enlarged CSF space with visible enlargement of the interhemispheric space in addition to the extracerebral space.
  3 = more substantially enlarged global subarachnoid space with visible cerebrospinal fluid between many gyri as well as interhemispheric and extracerebral.

6.8 Visit 5, Follow-Up with 2 years (FU2)

This first FU with almost 2 years is mandatory for all VLBW infants. The Bayley Scales III of Infant Development (BSID-III) will be performed and a final score similar to the intelligence quotient (IQ) with a mean of 100 points will be recorded and SD from mean. The IQ with 2 years is a secondary outcome parameter together with others.

The BSID-III includes the following items, scores (including subscores) and questions, which will be recorded in the eCRF:

- Cognitive, language (receptive and expressive communication), motor (fine and gross motor)
- Sensory-motor-neurological findings during exam
- Cerebral palsy (CP), spastic, dyskinetic or ataxic, degree of CP
- Any other neurodevelopmental abnormalities
- Any therapy, including medical, physical, early intervention, occupational, child psychiatry, others.
- Visual problems, Lang test, hearing problems
- Rehospitalisation during 1st year of life, if yes, specification of main reason
- Surgery for ROP or VP shunt

Additional parameters recorded will be:

- Date of FU2 and place of FU2
- Age at assessment (corrected)
- Weight (g), SD from mean weight.
- Head circumference (cm), SD from mean length.
- Length (cm), SD from mean length.

Course of haemorrhage:

Posthaemorrhagic hydrocephalus, graded 0-3:

- 0) Restitution ad integrum – no remaining lesions, date of last follow up
- 1) Persisting posthaemorrhagic hydrocephalus without any drainage
- 2) Persisting posthaemorrhagic hydrocephalus with repetitive but transient csf-drainage, indicate if LP, or Rickham, or both and indicate for how long
- 3) Posthaemorrhagic hydrocephalus with permanent csf-drainage, e.g. VP-Shunt

Convulsions:

- If present and/or any antiepileptic therapy, if yes specify treatment including duration

Other abnormalities or medications related to haemorrhage

- if yes specify

A 2 years composite outcome parameter will include death before a corrected age of 2 years or survival with 1 or more of the following: any motor impairment, cognitive impairment (IQ < 85), behavior problems such as attention disorder, poor general health, severe hearing loss, and bilateral blindness.

General health at 2 years will be stratified in normal and poor. Poor is defined as 1 or more of the following: need for supplemental oxygen, positive airway pressure, feeding through a tube or intravenously, seizures occurring more frequently than once per month, or a recent admission to an intensive care unit for complications resulting from a neonatal morbidity.

Socioeconomic status at 2 years will be calculated according to Largo RH et al. [62] by means of a six-point score of both paternal occupation and maternal education. The lowest possible SES score is 1, the highest 12. Socioeconomic status has been shown to affect cognitive development and therefore is an important confounder, which should be balanced in the EPO group and the placebo group.

6.9 Visit 6, Follow-Up with 5 years (FU5)

This second FU with 5 years is on the way to become mandatory for all VLBW infants. The Kaufman-ABC test will be performed and a final score similar to the intelligence quotient (IQ) with a mean of 100 points will be recorded and SD from mean[57]. The IQ with 5 years is the main outcome at 5 years and the primary study outcome at all.

The K-ABC includes the following items, scores (including subtests):

- Subtests: Atlantis, concept development, repeat numbers, rover, vocabulary, symbols, triangles, row of words, complete patterns, riddles
- Alternative subtests: Atlantis (called up after interval), symbols (called up after interval)
- Scales (sequential, simultaneous, learning, knowledge)
- Composite Score
- Sensory-motor-neurological findings
- Any therapy: physical, early intervention, occupational, speech & language, psycho-motor, child psychiatry, other
- Visual and hearing, “Zürcher Neuromotorik” including block components (purely motor, pegboard, dynamic balance, static balance)

Additional parameters recorded will be:

- Date of FU5 and place of FU5
- Age at assessment (corrected)
- Weight (g), SD from mean weight.
- Head circumference (cm), SD from mean length.
- Length (cm), SD from mean length.

Course of haemorrhage until 5 years:

Posthaemorrhagic hydrocephalus, graded 0-3:

- 0) Restitutio ad integrum – no remaining lesions, date of last follow up
- 1) Persisting posthaemorrhagic hydrocephalus without any drainage
- 2) Persisting posthaemorrhagic hydrocephalus with repetitive but transient csf-drainage, indicate if LP, or Rickham, or both and indicate for how long
- 3) Posthaemorrhagic hydrocephalus with permanent csf-drainage, e.g. VP-Shunt

Convulsions:

- If present and/or any antiepileptic therapy, if yes specify treatment including duration

Other abnormalities or medications related to haemorrhage

- if yes specify

Children will be classified at 5 years as either being normal or having minor or major impairment according to the overall results of the tests as summarized:

Normal development: All of the following:

- Normal neurological evaluation, IQ > 84
- No neurodevelopmental deficits

Minor impairment: One or more of the following problems:

- Subnormal cognitive abilities (IQ, 70-84)
- Gross and fine motor activity deficits
- Disorders of language development
- Visual and auditory deficiencies
- Attention disorders
- Abnormal socioemotional development

Major impairment: One or more of the following problems:

- Cerebral palsy
- Intellectual disability (US mental retardation) with IQ < 70
- Bilateral Blindness (Bilateral blindness is defined as a corrected visual acuity less than 20/200 in the better eye)
- Severe hearing loss (Severe hearing loss is defined as the prescription of hearing aids or cochlear implants)
- Intractable epilepsy (seizures occurring more frequently than once per month)

A 5 years composite outcome parameter will include death before a corrected age of 5 years or survival with 1 or more of the following: any motor impairment, cognitive impairment (IQ < 85), behavior problems such as attention disorder, poor general health, severe hearing loss, and bilateral blindness.

General health at 5 years will be stratified in normal and poor. Poor is defined as 1 or more of the following: need for supplemental oxygen, positive airway pressure, feeding through a tube or intravenously, seizures occurring more frequently than once per month, or a recent admission to an intensive care unit for complications resulting from a neonatal morbidity.

Socioeconomic status at 5 years will be calculated according to Largo RH et al. [62] by means of a six-point score of both paternal occupation and maternal education. The lowest possible SES score is 1, the highest 12. Socioeconomic status has been shown to affect cognitive

development and therefore is an important confounder, which should be balanced in the EPO group and the placebo group.

6.10 Addendum: Determination of blood and csf biomarkers

In all patients serial blood measurements are medically needed irrespectively of this trial. At three time points left-overs or additional 0.5 ml from blood measurements will be used to determine three blood biomarkers, copeptin, neurofilament light chain and doublecortin.

Time points: 1) Before first EPO medication usually at DOL 5-8.
2) At visit 2 (DOL 28)
3) At visit 3 (PMA 36)

If a patient develops posthaemorrhagic hydrocephalus and csf-drainage is required, then an additional 0.5 ml or left-over from medically needed blood measurements next to and ideally before csf-drainage will be drawn in EDTA plasma tubes and used to determine the above mentioned biomarkers. Left-overs from csf will be used to determine doublecortin in csf.

Blood sampling for blood and csf biomarkers will be performed only at larger study sites, including Zürich, Lausanne, Basel, Berlin and Vienna. In all cases no extra puncture will be done for the biomarkers and there must be no note as protocol deviation if blood sampling for the biomarkers was impossible at a certain time point.

Background: This trial enrolls infants with IVH grade II-IV of whom some develop posthaemorrhagic hydrocephalus. Both IVH and posthaemorrhagic hydrocephalus may result in subtle neuronal damage, which cannot be quantified by cUS in contrast to MRI. Neurofilaments (Nf) are specific scaffolding proteins of neurons and their quantification in serum serves as biomarker for axonal injury e.g. in neurodegenerative diseases (1). Thus, we hypothesize, that the degree of neuronal damage determined by MRI at expected date of delivery correlates with serum Nf concentrations in the weeks of life before. Posthaemorrhagic hydrocephalus leads to progressive intracerebral brain pressure and concomitant neuronal damage. Csf drainage is the only therapy but it is unclear when to commence. The stable surrogate marker of arginine vasopressin copeptin is increased in peripheral blood upon increased brain pressure and may therefore be a diagnostic adjunct to optimize the onset and duration of csf drainage (2). Doublecortin is a marker of juvenile neurons and is released to csf where it can be determined as a marker of neuronal remodeling (3).

Plasma and csf will be stored at each study site at -20°C and will be transferred to the University of Basel Children’s Hospital on dry ice for analyses in batches.

After completing study analysis remaining biologic samples will be disposed.

References:

1) Serum neurofilament light chain in early relapsing remitting MS is increased and correlates with CSF levels and with MRI measures of disease severity. Kuhle J, Barro C, Disanto G, Mathias A, Soneson C, Bonnier G, Yaldizli Ö, Regeniter A, Derfuss T, Canales M, Schluep M, Du Pasquier R, Krueger G, Granziera C. Mult Scler. 2016 Oct;22(12):1550-1559.
2) Role of vasopressin and its antagonism in stroke related edema. Ameli PA, Ameli NJ, Gubernick DM, Ansari S, Mohan S, Satriotomo I, Buckley AK, Maxwell CW Jr, Nayak VH, Shushrutha Hedna V. J Neurosci Res. 2014 Sep;92(9):1091-9.
3) NGF, DCX, and NSE upregulation correlates with severity and outcome of head trauma in children Chiaretti A, Barone G, Riccardi R, Antonelli A, Pezzotti P, Genovese O, Tortorolo L, Conti G. Neurology. 2009 Feb 17;72(7):609-16.

7 SUBJECT SELECTION AND WITHDRAWAL

7.1 Inclusion Criteria

Patients fulfilling all of the following inclusion criteria may be enrolled in the study

- Infants born before 32 weeks of gestational weeks and/or 1’500 g of birth weight
- IVH grade II-IV
- Chronological age < 9 days of life
- Informed written parental consent

7.2 Exclusion Criteria

The presence of any one of the following exclusion criteria will lead to exclusion of the subject:

- Genetically defined syndrome
- Severe congenital malformation adversely affecting life expectancy and/or neurodevelopment
- A priory palliative care
- Unlikely to participate at 5-year follow-up examination

7.3 Subject Recruitment and Screening

As only VLBW infants are eligible with an additional event to be diagnosed after birth (IVH grade II-IV), recruitment starts after birth. This is in contrast to the very recently for recruitment completed EPO-trial (www.clinicaltrials.gov; NCT00413946,[22]) in which parents were asked before birth as first administration of study dose is immediately after birth (3 h).

7.4 Early Withdrawal of Subjects

Subjects who discontinued the study will be replaced if recruitment is ongoing. Once recruitment is completed, no subject replacement is possible.

7.5 Criteria for Early Withdrawal of Subjects

Withdrawal of parental consent.

7.5.1 Data Collection and Follow-up for Withdrawn Subjects

If data are missing, e.g. not collected, they can be included in final analysis. We believe that few missing data are acceptable and are not influencing the study results. In case of subject withdrawal all data collected until withdrawal will be saved. After completing study analysis data of withdrawn subjects will be anonymized ~~unless parents explicitly abstain from making data anonymous~~.

To optimize data quality and to minimize queries by the monitors of the CTC Zürich, the local PIs will be supported in data input by the study staff, especially by the study nurse and by the co-investigator. Both, the study nurse and the co-investigator will visit each study site at least once and will help with data input in the eCRFs especially on request in case of problems.

To increase follow up rate and minimize drop outs, parents of participating subjects will receive financial compensation if necessary for participation in the visits after discharge, visit 4 (brain MRI), visit 5 (FU2) and visit 6 (FU5). There will be no regular amends. But if parents have to take off from work to participate visit 4 to 6 and/or complain travel expenses, the sponsor will reimburse these upon request.

7.6 Early Termination of Study

The Sponsor may terminate the study prematurely according to, see below some examples, which in any case would cause subsequent notification requirement (KlinV Art 38):

- insufficient patient recruitment
- when the safety or benefit of the subjects is doubtful or at risk, respectively
- alterations in accepted clinical practice that make the continuation of a clinical trial unwise
- reaching a positive or negative statistical end point earlier than anticipated

8 TREATMENTS

8.1 Treatments to be Administered

Subjects will receive at 5 times an intravenously administration of either 2’000 IU EPO/kg or placebo. Both, the verum and the placebo are a clear liquid of NaCl 0.9%. There will be no possibility to differentiate between verum or placebo by eyes, tasting or labelling. Drugs will be prepared as 1.5 ml pre-filled syringes by hospital pharmacy upon individual request. In detail, the Kantonsapotheke Zürich (KAZ) will supply the study sites in Zürich, Aarau, Basel, Chur, St. Gallen and Lausanne; the Spitalpharmazie of the Inselspital Bern will supply the study site in Bern. In Germany, the pharmacy of the University hospitals in Berlin (Charité) will supply the respective study site. In Austria, the pharmacy of the University hospital in Wien will supply the study site in Wien.

8.2 Identity of Investigational Product(s)Treatment, test drug

Recombinant human EPO will be purchased from Roche, Switzerland. The brand name is Recormon. A 1.5 ml solution will be prepared by hospital pharmacy upon individual request. Administration is once daily starting at DOL 5 -2/+3 (T1), 24 ± 2 hours after first administration (T2), 48 ± 2 hours after first administration (T3), then ~~10~~ 9 days after first administration (T10), and finally 16 days later (T17). A single dose consists of 16.7 µg (2000 IU) human erythropoietin per kilogram (kg) body weight dissolved in 1 ml NaCl 0.9%. It will be given intravenously within 2-3 minutes. The maximal dose is 25 µg (3000 IU) for infants weighing 1.5 kg or more.

Treatment, placebo

A 1.5 ml solution will be prepared by hospital pharmacy upon individual request. NaCl 0.9% will be administered according the same schedule as described for the test drug. A single dose consists of 1.5 ml NaCl 0.9% per kilogram body weight.

Dosage

The dosage of the study drug (investigational medicinal product, IMP) is calculated on the basis of body weight. Birth weight has to be used until the actual weight is higher. Then the actual weight has to be used. Infants with more than 1,5 kg receive maximal dosage of 3000 IU.

8.3 Method of Assigning Subjects to Treatment Groups

EPO as a verum is compared to NaCl 0.9% as a placebo 1:1. Randomization lists for block randomization will be performed for each participating study cite separately by the CTC Zürich.

8.4 Treatment Compliance

Study treatment will be prescribed by the local study doctor and administered by the nurse responsible for caring the participating infant.

8.5 Prior and Concomitant Therapy

The study treatment is an add-on treatment without any adaptations, restrictions or permissions of regular therapy. Any concomitant medication that is medically indicated for a patient will be allowed within the study.

There is one exception, patients enrolled in this EpoRepair study are not allowed to receive EPO for other purposes, e.g. for prevention or treatment of preterm anaemia. Among the participating centres only two centre, the Inselspital in Bern and the Kantonsspital in Chur, administer EPO regularly in VLBW infants starting at 4 to 6 weeks of age to prevent late transfusions of packed red cells. As all medications have to be prescribed individually, subjects enrolled in the EpoRepair study will not receive a prescription of EPO for treatment of anaemia. The local PIs of Bern and Chur will control this together with the study nurse and the co-investigator.

As outlined in the background section, alternative strategies have been developed to minimize blood loss and are successfully implemented in all other centres, including delayed cord clamping, minimizing blood-based laboratory diagnostics and early start in iron supplementation.

8.6 Packaging, Labelling and Supply

The packaging, blinding, labelling and storage of the medicine at al pharmacy sites conforms to the HMG, according to a randomisation list that is only known to the CTC Zürich as a whole and to the local pharmacy sites in part.

The individual pre-filled syringes will be allocated unequivocally by their labelling (centre, patient number). One vial will contain 2000 IU of EPO per ml and in total 3000 IU = 1.5 ml, this is the maximal amount per medication.

Each study site will order the medicine only patient-related and when a medication is scheduled. There will be no long-term storage, the medicine has to be administered within 7 days (shelf life). Adequate refrigeration (+2 to + 8°C) must be provided during short-term storage at the individual centres. Unused samples must be stored by the individual centres until the study monitor will have controlled and released the unused samples. Afterwards they can be disposed. Disposal of samples must be documented in a disposal protocol (included in the investigator's file).

Drug supply will be done as follows: The Kantonsapotheke Zürich (KAZ) will supply the study sites in Zurich, Aarau, Basel, Chur, Lausanne, and St. Gallen; the Spitalpharmazie of the Inselspital Bern the study site in Bern. In Germany, the Clinical Research Organization (CRO) of the University hospital in Berlin (Charité) will supply the respective study site. In Austria, the pharmacy of the University hospital in Wien will supply the study site in Wien.
The supply from Zurich to Aarau, Basel, Chur, Lausanne, and St. Gallen is organized as follows: After preparation of the study drug by the Kantonsapotheke Zurich, the study drug will be put in a transport cooling box, with guaranteed stability of temperature between 2 and 8°C for 24 hours (Neopor, STOROpack). Each box contains a temperature logger (Q-tag, Berlinger), which is used to monitor temperature during transport. After delivery the recipient at the local study site will charge the logger data electronically and will safe the file for study monitoring. The study drug transport will be done by Swiss-Express Mond (Die Post) with personal pickup in the afternoon and delivery next morning.

Upon receipt of the study treatment supplies, an inventory must be performed and a drug receipt log filled out and signed by the person accepting the shipment. It is important that the designated study staff counts and verifies that the shipment contains all the items noted in the shipment inventory. Any damaged or unusable study drug in a given shipment (active drug or comparator) will be documented in the study files.

Regular study drug reconciliation will be performed to document drug assigned, drug consumed, and drug remaining. This reconciliation will be logged on the drug accountability form, and signed and dated by the study team.

8.7 Blinding

The study is randomised on a double-blind basis. This means that the attending investigators do not know whether they are using the placebo or the verum. Randomisation is performed by the CTC Zürich on the basis of randomisation lists. Randomization lists for block randomization will be performed for each participating study cite separately by the CTC Zürich.

In the event that deblinding should be required subsequent to an individual patient's withdrawal from the study, this will be possible by immediate notification of the responsible pharmacy or the CTC Zürich, with specification of the centre and randomisation number, within 24 hours.

In the event of deblinding, the PI has to be notified within a period of 24 hours. The PI will then take charge of further reporting as required by the GCP guidelines.

An Emergency Code Break will be available to each investigator. This Code Break should be opened only in emergency situations when the identity of the investigational product must be known by the investigator in order to provide appropriate medical treatment.

8.8 Storage Conditions

On each neonatology ward only short term storage is necessary, maximum 7 days. For this purpose adequate refrigeration (+2 to + 8°C) and protection from light will be performed in the refrigerator for medications for which limited access is given.

8.9 Study Drug Accountability

The study drug supplied on request to the local PIs will be kept in a secure, limited access storage area under the recommended storage conditions.

The local PIs will maintain accurate and adequate records including dates, lot number, quantities received, individual usage.

8.10 Return or Destruction of Study Drug

At the completion of the study, there will be a final reconciliation of drug shipped, drug consumed, and drug remaining. This reconciliation will be logged on the drug accountability form, signed and dated. Any discrepancies noted will be investigated, resolved, and documented prior to return or destruction of unused study drug.

9 STUDY PROCEDURES

All patients will be assessed with regard to inclusion/exclusion criteria (sections 7.1 and 7.2). If the criteria are met, the patient shall be included in the study after written informed parental consent has been obtained. The study medication will be obtained from the responsible pharmacy. It will be supplied on request.

9.1 Study-related Procedures

Procedures during NICU hospitalization

The following tests are standard procedures performed routinely in all VLBW infants and especially in those with IVH > grade 1 independent of enrolment in the study (medically indicated tests and examinations). The local PIs will take care that the following tests will be done completely:

- Minimal electronic monitoring includes heart rate (or pulse rate) with recommended alarm limits at 90 and 180 beats per minute, arterial saturation (pulse oximetry) with recommended alarm limits at 85% and 95%.
- Arterial blood pressure will be monitored continuously if there is an indwelling arterial line and intermittently by oscillometric measurement at least daily during the first weeks.
- Serial determination of Hb and Hct is part of standard care, in the first days of life daily, when infants are stable then at least weekly until discharge.
- Serial cUS assessment will be carried out on day 1, 3, and at day 7 and then every 14 days until 36 $^{0}/_{7}$ weeks postmenstrual age (or at discharge if discharged before) to detect intracranial haemorrhage, ventricular dilatation and white matter disease [7,19].
- MRI: There is clinical need to perform MRI in VLBW infants with severe IVH to determine precisely the extend of IVH especially if posthaemorrhagic hydrocephalus occurred and to predict outcome, which is important in counselling parents and managing follow-up. The access of each neonatology level III centre to MRI and the routine practice vary largely between centres. Also the timing of MRI is not standardized.
- Both eyes are examined by an ophthalmologist to detect retinopathy of prematurity (ROP). The severity of ROP will be graded according to the international classification of ROP.[63]
- Necrotising enterocolitis (NEC) is diagnosed by clinical signs (abdominal distension, bilious aspirates, and/or bloody stools) and either pneumatosis intestinalis, hepatobiliary gas, or free intraperitoneal air on abdominal x-ray or at laparotomy. Severity of enterocolitis will be graded according to presence and severity of complications.
- Septicaemia is diagnosed by clear clinical, radiological, or biochemical evidence of infection and at least one microbiologically relevant positive blood culture.
- Persistent ductus arteriosus (PDA) is diagnosed clinically or with echocardiography and documented only if drug treatment (indomethacin or ibuprofen) or surgical ligation is required.
- Weight and head circumference are recorded at birth, at 7 to 10 days, at 36 $^{0}/_{7}$ postmenstrual weeks, at 24 months and 5 years corrected age.
- Placental histology is regularly done in infants born very preterm. It identifies prenatal risks such as amnion infection and placental malfunction.
- Adaptation after birth will be documented with the Apgar score at 1, 5 and 10 minutes.
- The Clinical Risk Index for Babies (CRIB). The CRIB is a simple tool for assessing neonatal risk based on the variables gestational age, birth weight, congenital malformations, lowest and highest appropriate FiO2 and worst base deficit within the first 12 hours of life.[64]

9.2 Study-specific Procedures

Apart from the administration of study medication there is no procedure within this study which is not part of routine management of VLBW infants, including MRI in infants suffering from severe IVH. As the timing of MRI is not standardized the study-specific requirement is to perform MRI at term equivalent age, visit 4.
All items recorded by MRI are given in visit 4, section 6.7. In principal, the MRI for all VLBW infants will be performed at the site of hospitalisation. If discharge home occurred before time point of visit 4, VLBW infants will be invited to come together with their mother to the MRI, which will be performed in natural sleep. This occurs usually after feeding and is sufficient to keep the infant quiet enough for MRI. In general, no medications will be given and no line will be installed. This procedure has been established during the first EPO trial at the study centre in Zurich (USZ). However, as the MRIs will be performed not only at the USZ, other study sites may have different approach for keeping the infant quiet during MRI. These specific in-house approaches are acceptable within this study and are in the responsibility of the local doctors.

9.3 Sample preparation, storage, and shipment

There is no collection of study samples planned, e.g. blood or tissue. However, the cUS and MRI files will be saved together with patient records.

Please see also chapter 6.10 for an addendum

10 SAFETY

The Sponsor’s SOPs provide more detail on safety reporting.

10.1 Definition of (Serious) Adverse Events

Adverse events

Adverse events (AEs) are defined as any untoward medical occurrence in a patient or clinical investigation subject administered a pharmaceutical product and which does not necessarily have a causal relationship with this treatment. An AE can therefore be any unfavourable and unintended sign (including an abnormal laboratory finding), symptom, or disease temporally associated with the use of a medicinal study product, whether or not related to the medicinal study product. An AE may also consist of a new disease, an exacerbation of a pre-existing illness or condition, a recurrence of an intermittent illness or condition, a set of related signs or symptoms, or a single sign or symptom.

AEs observed by the investigator and/or reported by the caregivers or parents must be reported in the eCRF during the entire study period, the period of time from the first drug administration until discharge home.

For all AEs, sufficient information will be pursued and/or obtained so as to permit an adequate determination of the outcome of the event (i.e., whether the event should be classified as an SAE) and an assessment of the casual relationship between the AE and the investigational drug or study treatment(s).

Whenever available, the underlying disease or condition for which a therapeutic or diagnostic procedure is required should be reported as the AE term. Surgeries or other invasive procedures that had already been planned prior to the start of the study do not have to be documented as AEs. These planned procedures will be recorded in the eCRF by the investigator at the baseline visit. It is not important if the condition was known before enrolment, only if the procedure was planned before.

This trial enrols highly vulnerable and largely immature neonates who are born very preterm and thus are unable to survive without intensive care support. Most organ functions are physiologically immature in the neonatal period. The degree of immaturity is aggravated due to prematurity. Prematurity is characterized by the absence of abilities a healthy born infant has a priori when born at term. These abilities include but are not limited to body water homeostasis, body temperature maintenance, skin vulnerability, stable breathing and heart rate, drinking, digestion, liver function, and host defense (Guideline on the investigation of medicinal products in the term and preterm neonate, EMA, June 2009, Doc. Ref. EMEA/536810/2008). Therefore, the preterm body shows a large number of different symptoms which are characteristic hallmarks of immaturity and which disappear gradually alongside maturation over a timespan of many weeks, see also the below given short list of symptoms typically present in all very preterm infants (further information is given e.g. in the textbook edited by Maier RF and Obladen M, Neugeborenenintensivmedizin – Evidenz und Erfahrung, Springer 2011).

In addition to this immaturity there are various signs, particularly affecting the skin, owing to intensive care and fixation of life saving devices mainly on the skin by plasters, stripes and patches. These devices include but are not limited to breathing tubes, feeding tubes, intravenous lines, electrodes and sensors. As a result, mainly the immature skin shows transient signs of fixation and positioning as listed below.

In conclusion of both, large immaturity and intensive care, all very preterm infants are characterized by a variety of signs which are typical and expected in this population and must not be considered as an AE. To clarify this, there is in addition to the list of immaturity signs and intensive care signs a third list providing samples of AEs, which comprise common deteriorations of very preterm infants but which are not regularly expected and may indicate

therefore any unfavourable and unintended sign, symptom, or disease temporally associated with the use of a medicinal study product, whether or not related to the medicinal study product.

1) Examples of immaturity signs not qualifying as AE:

- **Apnea and oxygen desaturations** occurring in all very preterm infants due to immaturity of brain and lung and are treated with caffeine, oxygen and if severe with respiratory support.
- **Episodes of bradycardia** occur in almost all very preterm infants often in combination with apnea and oxygen desaturation.
- **Edema** occurs in almost all very preterm infants after birth due to kidney immaturity and usually disappears within few weeks. Edema may affect the whole body if severe or only typical regions, such as eyes, hands and feet.
- **Jaundice** is usually present in the first and second week of very preterm infants due to liver immaturity.
- **Skin barrier damage** is the consequence of immature skin and may occur by simply lying on the skin or touching it. Skin of very preterm infants is highly vulnerable and in regard to treatment similar to that of burn victims.
- **Gastric residuals** are often present and mainly transient in the first weeks of life.
- **Hypothermia or hyperthermia** following inappropriate incubator temperature.

2) Examples of intensive care signs not qualifying as AE:

- **Local redness of the skin** occurs always after removal of e.g. plasters, sensors, strips and is visible for few hours but sometimes for days.
- **Local trauma of the skin including local haematoma** is unavoidable when performing a heel prick, vein puncture or other medically needed invasive procedures braking skin integrity. Afterwards the damage is visible for few hours but sometimes for days.

3) Examples of AEs:

- **Anaemia** needing blood transfusion (RBC-transfusion)
- **ROP** Grade: Mild: Grade 1 → **AE**
  Moderate: > Grad 1 or plus disease → **AESI,** see below
  Severe: needing intervention → **SAE**, see below
- **BPD** Grade: Mild: no AE
  Moderate: O2 < 30% mit 36. PMA → **AE**
  Severe: O2 ≥ 30% mit 36. PMA → **AE**
  Prolongation of hospitalization due to BPD beyond EDD (requiring any respiratory support because of BPD) → **SAE**
- **Posthaemorrhagic ventricular dilatation**:
  Moderate if ventricular dilation is > 97 centile → **AE**
  Severe if ventricular dilation is > 4 mm above 97th centile. → **AE**
- **Patent ductus arteriosus:** if medical treatment is required (not preventive) and/or surgical ligation
- **Incorrect drug applications**

Adverse events of special interest

As adverse events of special interest (AESIs) we defined ROP grade 2 or > 2 or Plus disease without intervention. Although in the preventive EPO study, section 4.6, no differences were found between EPO and placebo treated infants regarding ROP, there is still some concern regarding the EPO treatment in preterm infants regarding ROP. To address this issue and to increase alertness within this EpoRepair trial we defined all moderate stages of ROP as AESI which will be reported to the sponsor according to the same rules as SAEs.

Serious adverse event

A SAE is any untoward medical occurrence that at any dose

• results in death,
• is life-threatening,
• requires subject hospitalization or prolongation of current hospitalization,
• results in persistent or significant disability/incapacity, or
• any important medical event and any event which, though not included in the above, may jeopardise the subject or may require intervention to prevent one of the outcomes listed above.

Any other medically important condition that may be not immediately life-threatening or results in death or hospitalization but may jeopardize the subject or may require intervention to prevent one of the outcomes listed above should also usually (i.e. based on medical and scientific judgment) be considered serious. For example: intensive treatment at home for allergic bronchospasm; certain laboratory abnormalities (e.g. blood dyscrasias); convulsions that do not result in hospitalisation; development of drug dependency or drug abuse.

Examples of SAEs:

- **Sepsis**, if blood culture is positive
- **NEC and/or perforation**, If Bell stage ≥ IIa (pneumatosis proven) and/or perforation even without signs of NEC (e.g. Focal Intestinal Perforation, FIP)
- **ROP**, if intervention is required
- **BPD**, if severity leads to prolongation of hospitalization beyond EDD
- **Death**

Suspected unexpected serious adverse reaction (SUSAR)
A serious adverse reaction, the nature or severity of which is suspected to be not consistent with the applicable IB.

Safety Signals

All suspected new risks and relevant new aspects of known adverse reactions that require safety-related measures.

As this is a phase III study details are given in the IB.

10.2 Recording of (Serious) Adverse Events

The well-being of the subjects will be ascertained by medical caregiver when subjects are hospitalized and the parents when subjects are discharged. Clinical investigators and ultimately the protocol PI have the primary responsibility for AE identification, documentation, grading, and assignment of attribution to the investigational agent/intervention.

All observed or volunteered adverse drug events (serious or non-serious) and abnormal test findings, regardless of treatment group or suspected causal relationship to the investigational drug or study treatment(s) will be recorded in the patient file and subsequently in the eCRF.

Recording will be done from first study treatment DOL 5 (-2/+3 days) until discharge home. As an exception the defined AEs (ROP grade >1 or Plus diseases, BPD if classified as severe at 36 PMA, and anaemia) will be recorded without time limit.

AEs or abnormal test findings felt to be associated with the study treatment(s) will be followed until the event (or its sequelae) or the abnormal test finding resolves or stabilizes at a level acceptable to the investigator.

An abnormal test finding will be classified as an AE if one or more of the following criteria are met:

- The test finding is accompanied by clinical symptoms.
- The test finding necessitates additional diagnostic evaluation(s) or medical/surgical intervention; including significant additional concomitant drug treatment or other therapy

Note: simply repeating a test finding, in the absence of any of the other listed criteria, does not constitute an AE.

- The test finding leads to a change in study dosing or discontinuation of subject participation in the clinical study.

All AEs, serious and non-serious, will be fully documented in the appropriate eCRF. For each AE, the investigator will provide the onset, duration, intensity, treatment required, outcome and action taken with the investigational product.

10.3 Assessment of (Serious) Adverse Events

The investigator will promptly review documented AEs and abnormal test findings to determine if

- the abnormal test finding should be classified as an AE,
- if there is a reasonable possibility that the AE was caused by the investigational drug or study treatment(s), and
- if the AE meets the criteria for an SAE.

The intensity of an AE will be assessed by the investigator as being

- mild (hardly noticeable, negligible impairment of well-being),
- moderate (marked discomfort, but tolerable without immediate relief), or
- severe (overwhelming discomfort, calling for immediate relief).

The assessment of causality to the study drug by the investigator is done according to the following definitions:

Unrelated	• The event started in no temporal relationship to medicinal product applied and<br>• The event can be definitely explained by underlying diseases or other situations.
Related	• The event started in a plausible temporal relationship to medicinal product applied and<br>• The event cannot be definitely explained by underlying diseases or other situations.

10.4 Reporting of Serious Adverse Events and other Safety Related Events

SAEs and other Safety Related Events (definition under Reporting of Safety signales) have to be reported to the Sponsor **within 24 hours** by the PI or the Investigator.

Such a report must be done by an appropriate entry in secuTrial, which must be completed as SAE to trigger automatically an email alert to the Sponsor. If secuTrial access is not possible or any uncertainty has arisen, reporting should be done by e-mail buh@usz.ch.

The Investigator is responsible for SAE reporting to the EC according to the following details:

- Reporting to EC any SAE which resulted in death:
  - without delay, and no later than 7 calendar days.
- Reporting to EC of fatal SAEs if evaluated as “suspected”, “unexpected” and “drug related” (SUSAR)
  - without delay and no later than 7 calendar days following awareness that event meets criteria for an SUSAR.
- Reporting to CEC of non-fatal SAEs if evaluated as “suspected”, “unexpected” and “drug related” (SUSAR):
  - promptly and no later than 15 calendar days following awareness that event meets criteria for a SUSAR.
- All other SAEs will be summed up in the annual safety update report.

The Sponsor is responsible for SAE reporting to Swissmedic according to the following details:

- Compliance with the regulatory requirements of Swissmedic regarding prompt reporting of unexpected SAEs for which a causal relationship with the study drug cannot be ruled out.
- Reporting to Swissmedic of fatal SAEs if evaluated as “suspected”, “unexpected” and “drug related” (SUSAR):
  - **without delay** and no later than **7 calendar days** following awareness that event meets criteria for a SUSAR;
- Reporting to Swissmedic of non-fatal SAEs if evaluated as “suspected”, “unexpected” and “drug related” (SUSARs):
  - **promptly** and no later than **15 calendar days** following awareness that event meets criteria for a SUSAR.
- Sending Annual Safety Reports (ASR), starting one year after the date of notification to Swissmedic. These reports should contain:
  - A concise critical summary of the safety profile of the drug studied as well as the safety issues that have arisen;
  - A listing of all SUSARs that have occurred in Switzerland and at international level (if applicable);
  - Ideally all adverse drug reactions at international level.
  - The accompanying letter provided with the Annual Safety Report should contain a short summary of the status of the clinical trial in Switzerland (number of centres open/closed, number of patients recruited/recruitment closed, and number of SAR/SUSAR.

Reporting of Safety Signals

All suspected new risks and relevant new aspects of known adverse reactions that require safety-related measures, i.e. so called safety signals, must be reported to the Sponsor-Investigator within 24 hours. The Investigator must report the safety signals within 7 days to the local Ethics Committee (local event via local Investigator) and the Sponsor to Swissmedic, respectively.

The Sponsor-Investigator must immediately inform all participating Investigators about all safety signals. The other in the trial involved Ethics Committees will be informed about safety signals in Switzerland via the Sponsor-Investigator.

An unexpected SAE refers to any AE, the nature or severity of which is not consistent with the applicable product information as given in the current IB.

In order to monitor continuously safety of the IMP, the sponsor will release monthly an export of all SAEs captured by the Secutrial system, which he will evaluate critically and file his evaluation in the TMF.

10.5 Follow up of (Serious) Adverse Events

Subjects terminating the study (either regularly or prematurely) with

- reported ongoing SAE, or
- any ongoing AEs of laboratory values or of vital signs being beyond the alert limit

will return for a follow-up investigation. This visit will take place up to 30 days after terminating the treatment period. Follow-up information on the outcome will be recorded on the respective AE page in the eCRF. All other information has to be documented in the source documents. Source data has to be available upon request.

In case of subjects lost to follow-up, efforts should be made and documented to contact the subject to encourage him/her to continue study participation as scheduled.

All new SAE that the investigators will be notified until discharge home have to be reported in appropriate report forms and in the eCRF.

Follow-up investigations may also be necessary according to the investigator’s medical judgment even if the subject has no AE at the end of the study. However, information related to

these investigations does not have to be documented in the eCRF but must be noted in the source documents.

10.6 Data Safety Monitoring Board

The Data Safety Monitoring Board, which is called according to international guidance ‘Data Monitoring Committee’ (DMC) consists of two members of the Swiss Research Network of Clinical Pediatric Hubs (SwissPedNet) and one biometrician. The DMC will monitor the AEs and can suggest modifications or stop the trial if the risk/benefit ratio for the patients is significantly changed and the pursuit of the trial may harm the patients. The DMC meets or convene by telephone conference or by e-mail correspondence at least once per year and after an interim analysis, when the first 50 patients passed visit 2. The sponsor will receive a report from the DMC after each meeting regarding study continuation. All other issues from the not public meetings of the DMC will be kept under strict confidentiality, including written protocols of DMC meetings and unblinded patient data. The report from the DMC, as well as the minutes of the discussion between sponsor and DMC and any potential consequences from this will be communicated in writing to all study centres.

10.7 Addendum only for study centres in Switzerland

If a study participant has to be transferred to another hospital e.g. because of limitations of capacity at the study centre, specific actions are required which are all listed in a specific SOP-08 “Verlegung”. Such hospitals are considered as satellite study centres and patient transfer occurs almost exclusively after visit 2, when study treatment is completed. Thus, satellite study centres are never involved in patient recruitment and enrolment and usually not in study treatment. In case of urgent medical need e.g. because of limitations of capacity at the study centre, exceptions are possible but subject to strict regulatory requirements as specified in the SOP-08 “Verlegung”.

11 DATA QUALITY ASSURANCE

The Sponsor is implementing and maintaining quality assurance and quality control systems with written SOPs and WIs endorsed by the CTC Zürich, to ensure that trials are conducted and data are generated, documented (record), and reported in compliance with the protocol, GCP, and applicable regulatory requirement(s).

The Sponsor is responsible to have written SOPs and WIs in place for the study and to provide those to all participating study sites. The local PIs at all sites must have a manual of the relevant SOPs and WIs for the study on site and are responsible for proper training of all involved study personal for the respective procedures.

Monitoring will be conducted by the CTC Zürich during the course of the study for quality assurance purposes.

11.1 Data evaluation

For quality assurance of outcome parameters, including MRI, FU2 and FU5, specific measures have been taken.

Ad cUS and MRI: Brain imaging will be done locally according to the medical requirements. The scoring according to the predefined and widely accepted scoring system for cUS and MRI as given in paragraph 6.2 and 6.7, respectively, will be done centralised under the responsibility of Dr. C. Hagmann, who is responsible within this study for MRI data analysis. For this purpose all cUS and MRI images will be sent on CD-Rom and scoring data will be captured in the eCRF. Ad FU2 and FU5: Both Follow-Ups will be done locally by the local professionals for child development as it is standard practice in Switzerland, Germany, and Austria. Independent follow-up commities in all three countries assure professional performance of FU2 and FU5 according to the specific tests. The results of the specific standardized tests, FU2 the Bayley Scales III of Infant Development (BSID-III) and FU5 the Kaufman-ABC test, will be captured in the eCRFs as described in paragraph 6.8 and 6.9, respectively.

11.2 Routine Monitoring

Regular monitoring visits at the investigator’s site prior to the start and during the course of the study will help to follow up the progress of the clinical study, to assure utmost accuracy of the data and to detect possible errors at an early time point. The Sponsor organises professional independent monitoring for the study.

All original data including all patient files, progress notes and copies of laboratory and medical test results must be available for monitoring. The data recording of the FU2 and FU5 visits is done electronically using the neonet computer interface (http://www.neonet.ch/en/neonatal-network/data-collection/). A print out of the FU2 or FU5 digital entry will be available as source data at the respective study site, signed and dated by the investigator. The monitor will review all or a part of the CRF/eCRFs and written informed consents. The accuracy of the data will be verified by reviewing the above referenced documents. The investigator's site will collaborate with the Clinical Trials Centre (CTC) of the University Hospital Zurich to ensure regular monitoring. According to the CTC's Monitoring SOP the extent and nature of monitoring activities based on the objective and design of the study will be defined in a study specific Monitoring Plan.

11.3 Inspections

A quality assurance inspection of this study will be conducted by the regulatory authority or IEC, respectively. The quality assurance inspector will have access to all medical records, the investigator's study related files and correspondence, and the informed consent documentation that is relevant to this clinical study.

The investigator will allow the persons being responsible for the audit or the inspection to have access to the source data/documents and to answer any questions arising. All involved parties will keep the patient data strictly confidential.

11.4 Specification of Source Documents

The following documents are considered source data, including but not limited to:

- SAE worksheets
- Nurse records, records of clinical coordinators, and
- Medical records from other department(s), or other hospital(s), or discharge letters and correspondence with other departments/hospitals, if subject visited any during the study period and the post study period.

Source data must be available at the site to document the existence of the study subjects and substantiate the integrity of study data collected. Source data must include the original documents relating to the study, as well as the medical treatment and medical history of the subject.

The following information (at least but not limited to) should be included in the source documents:

- Demographic data (age, sex)
- Inclusion and Exclusion Criteria details
- Participation in study and signed and dated Informed Consent Forms
- Visit dates
- Medical history and physical examination details
- Key efficacy and safety data (as specified in the protocol)
- AEs and concomitant medication
- Results of relevant examinations
- Laboratory printouts
- Dispensing and return of study drug details
- Reason for premature discontinuation
- Randomization number

12 STATISTICS

12.1 Statistical and Analytical Plans

There is presently no established medical therapy to ameliorate the neurological sequelae of IVH in VLBW infants.[14] The beneficial effects of EPO must be considered hypothetical (based on retrospective analyses) until confirmed or refuted by a RCT. Our trial will be a multicentre, placebo-controlled, prospective, randomised, double-blind (verum:placebo = 1:1) clinical study.
8 centres in Switzerland, 3 in Germany, and 1 in Austria participate in the study. The study will include female and male VLBW infants with IVH (grade II-IV) diagnosed by cUS at a chronological age < 8 d (more than 96% of all cases of IVH occur within the first 5 d of life). The primary endpoint is the IQ of the children at age of 5 years (continuous outcome) and in addition the binary endpoint normal development. Moreover, 4 secondary endpoints are planned: MRI outcome, cUS outcome, BSID-III, and drug safety (including mortality), – the first 3 outcomes have ordinal and continuous measures and the last outcome is binary. Based upon these design considerations, the study will be analyzed using t-tests, linear and logistic regression.

12.2 Endpoints and hypotheses

Primary endpoint:
Composite IQ (K-ABC or SON-R) at age of 5 years (continuous outcome)

Secondary endpoints:
Normal overall developmental outcome at age of 5 years will be a secondary end point (binary variable). Normal development is determined in a neurological assessment at 5 years of age, including intelligence quotient (K-ABC or SON-R). Normal development at 5 years is defined as the composite outcome of: Normal neurological evaluation, IQ > 84, and no neurodevelopmental deficits.

Other secondary endpoints:
Advanced MRI at 40 weeks postmenstrual age (continuous outcome)
cUS at DOL 28 and at PMA 36 weeks (ordinal and continuous outcome)
BSID-III at two years of age (continuous outcome)
Mortality (binary outcome)

Hypotheses:
The alternative hypothesis for all endpoints is improvement with regard to the endpoint in the group of EPO-treatment. The null hypothesis for all endpoints is no improvement with regard to the endpoint in the group of EPO-treatment compared to the control group.

The study hypotheses are one-sided – however, the tests will be performed 2-sided yielding corresponding confidence intervals and accordingly information about the direction of a potential difference between control and treatment group.

The significance level alpha is 0.05.

12.3 Planned Analyses

A descriptive analysis of the data will be performed providing a visual impression about the distributions of the outcomes in the different treatment groups and centres.

The statistical inference will be done on 3 levels:

1) a) Determining confidence intervals for the difference of IQ and other continuous outcomes between the 2 groups and p-values of the corresponding t-tests.
   b) Determining confidence intervals for risk differences and corresponding p-values for the binary outcomes.
2) We will adjust for study centres taking into account possible centre effects.

3) Using linear and logistic regression to adjust for additional relevant confounding variables (gestational age, child weight at birth, age of mother) having potentially a large impact on the outcome.
   For analyses of safety data by the SDMB, including an interim analysis when the first 50 patients passed visit 2, see paragraph 10.6. Here, mainly a descriptive analysis will be performed to get insight about the distributions of the safety relevant outcomes in the different treatment groups and centres

12.4 Handling of Missing Data

Multiple imputation will be used to adjust for missing outcomes due to death or dropout. In case of study cancellation irrespective of reason, all data collected will be saved as stated above.

12.5 Determination of Sample Size

Information about effect size and standard deviation of the primary endpoint (IQ outcome at age of 5 years) can only be derived from one observational study presented by Neubauer AP et al.[42] who investigated the effect of EPO-treatment of VLBW infants on composite IQ at 10 years.

Neubauer AP et al. included in their study all grades of IVH, also the lowest, IVH I°.[42] However, diagnosis of IVH I° may be missed or even overestimated in very preterm infants. Neubauer AP et al. reported on no difference in infants without IVH regarding composite IQ at 10 years between EPO-treated and untreated,[42] hence we decided to exclude infants with borderline diagnosis of IVH I° to avoid bias from misdiagnosis.[7]

Neubauer et al. reported for the relevant group of children who suffered IVH an effect size between EPO-treated (IQ=90.3) vs. untreated (IQ=67.0) of 23, standard deviation (SD) 21, $p < 0.01$. Given that the vast majority of children will remain on their 5-year IQ trajectory, and there is good agreement between classification of cognitive abilities at 5 and 11 years of age,[61] we used this continuous outcome parameter (effect size 23) for power analysis.

To determine the required sample size for the primary outcome we performed a power calculation for a 2-sample t-test assuming a significance level of alpha=0.05, SD=21 of the outcome, and a 1:1 randomization. As result we get as required total sample size n=28 (for both groups together), and n=38 for a power (1-beta) of 0.8 and 0.9, respectively.

When assuming a smaller effect size of 10 (instead of 23), a SD of 15 (instead of 21 because IVH °I will be excluded in our study group to avoid misdiagnosis leading to a sample which is less heterogeneous with regard to the primary outcome) and a power of 0.8, the required total sample size is n=72.

Based on the only available data of Neubauer et al. and the just mentioned reduction of the assumed effect size to 10 (instead of 23) and SD to 15 (instead of 21) we set the target sample size for visit 4, the secondary endpoint 'MRI at term equivalent age', at n=50 for EPO and n=50 for placebo. Given a 20% loss to follow up until 5-year, n=80 will meet the primary endpoint, visit 6. This number is in line with the result of the power analysis presented above. Hence, to achieve the target sample size for visit 4 of n=100 and an assumed early loss by about 15% due to e.g. death in this high-risk VLBW infant population, n=120 VLBW infants with IVH II-IV have to be enrolled in this trial.

According to data of the Bundesamt für Statistik (www.bfs.admin.ch) and the Swiss neonatology register (Minimal Neonatal Dataset, MNDS, www.neonet.ch) more than 800 very low birth weight (VLBW) infants were born each year since 2007 in Switzerland. The participating 8 Swiss neonatology level III units treat more than 80% of all VLBW infants in Switzerland (n>700 per year). To increase the number of eligible VLBW infants per year, the neonatology units of the University hospitals in Berlin (Charité) and Wien will contribute, treating together about 400 VLBW infants per year. Together, at all 9 study sites, more than 1000 VLBW infants will be assessed for eligibility each year.

As this trial offers a unique chance to receive a potentially beneficial treatment for IVH, the majority of parents are expected to be sympathetic to the trial (70%). Together, to achieve a target sample size of n=80 for the primary endpoint (visit 6) a total number of 1670 VLBW infants have to be assessed for eligibility:

- assessed for eligibility n=1'6700 VLBW infants → 12% have IVH grade 2-4
- presence of IVH II-IV n=200 VLBW infants (IVH II-IV) → 15% loss due to exclusion criteria
- asked for participation n=170 VLBW infants (IVH II-IV) → 30% no parental consent
- to be enrolled in trial n=120 VLBW infants (IVH II-IV) → 15% early loss (death)
- to be analyzed at visit 4 n=100 VLBW infants (IVH II-IV) → 20% loss at 5-year follow-up
- to be analyzed at primary endpoint (visit 6) n=80 VLBW infants (IVH II-IV)

The expected enrolment goal is 5 subjects per month at all study sites in Switzerland, Germany, and Austria over two years. If the enrolment goals will not met, remedial actions will be a prolongation of recruitment time until 100 VLBW infants reached the secondary endpoint 'MRI at term equivalent age'.

13 DATA HANDLING AND RECORD KEEPING

The study will strictly follow the protocol. If any changes become necessary, they must be laid down in an amendment to the protocol. All amendments of the protocol must be signed by the Sponsor and submitted to CEC and Swissmedic (KlinV Art 29 and 34).

The investigators will use electronic case report forms (eCRF), one for each enrolled study participant, to be filled in with all relevant data pertaining to the subject during the study. All subjects who either entered the study or were considered not-eligible or were eligible but not enrolled into the study additionally have to be documented on a screening log. The investigator will document the participation of each study subject on the Enrollment Log.

For data and query management, monitoring, reporting and coding an internet-based secure data base SecuTrial developed in agreement to the Good Clinical Practice (GCP) guidelines provided by the Clinical Trials Centre (CTC) Zurich will be used for this study. It is the responsibility of the investigator to assure that all data in the course of the study will be entered completely and correctly in the respective data base. Corrections in the eCRF may only be done by the investigator or by other authorised persons. In case of corrections the original data entries will be archived in the system and can be made visible. For all data entries and corrections date, time of day and person who is performing the entries will be generated automatically.

eCRFs will be kept current to reflect subject status at each phase during the course of study. Subjects will not to be identified in the *eCRF* by name. Appropriate coded identification (Subject Number) will be used without involving initials.

It will be assured that any authorised person, who may perform data entries and changes in the *eCRF*, can be identified. A list with signatures and initials of all authorised persons will be filed in the study site file and the trial master file, respectively.

Documented medical histories and narrative statements relative to the subject's progress during the study will be maintained. These records will also include the following: Copies of cUS images from visit 0, 2 and 3 and images from brain MRI which must be kept on file with the individual subject's *eCRF*.

In order to pseudo-anonymize all images, each study site will be reminded to blind patient name and birth date on all images prior to sending the images to the MRI specialist PD Dr. Hagmann for analysis. All study sites are encouraged to use the blinding option included in all patient information systems once images are burnt to CD-ROM.

The investigators assure to perform a complete and accurate documentation of the subject data in the *eCRF*. All data entered into the *eCRF* must also be available in the individual subject file either as print-outs or as notes taken by either the investigator or another responsible person assigned by the investigator. Essential documents must be retained for at least 10 years after the regular end or a premature termination of the respective study (KlinV Art. 45).

Any patient files and source data must be archived for the longest possible period of time according to the feasibility of the investigational site, e.g. hospital, institution or private practice.

Parents will be informed about all findings obtained by study-related and study-specific procedures including cUS and MRI. This communication with parents includes expected as well as unexpected findings especially by cUS or MRI.

14 CONFIDENTIALITY

The investigators are liable to treat the entire information related to the study and the compiled data strictly confidentially. Any passing-on of information to persons that are not directly involved in the study must be approved by the owner of the information.

Data generation, transmission, archiving and analysis of personal data within this study, strictly follows the current national legal requirements for data protection in Switzerland, Germany and Austria. Prerequisite is the voluntary approval of the subject’s parents given by signing the informed consent prior start of participation of the clinical trial.

Individual subject medical information obtained as a result of this study is considered confidential and disclosure to third parties is prohibited. Subject confidentiality will be further ensured by utilising subject identification code numbers to correspond to treatment data in the computer files.

Such medical information may be given to the subject's personal physician or to other appropriate medical personnel responsible for the subject's welfare, if the patient has given his/her written consent to do so.

Data generated as a result of this study are to be available for inspection on request by the monitors, by the CEC and the regulatory health authorities.

15 INSURANCE

Insurance for all patients enrolled in the study in Switzerland is covered by “Zurich-Versicherungs-Gesellschaft AG, Postfach, 8085 Zürich“ (Policy no.: 1099).

Insurance for patients enrolled in Germany and Austria will be taken separately.

Any damage developed in relation to study participation is covered by this insurance. So as not to forfeit their insurance cover, the subjects themselves must strictly follow the instructions of the study personnel. Subjects must not be involved in any other medical treatment trial without permission of the principal investigator (regular treatment excluded). The investigator must also be informed instantly, in the event of health problems or other damages during or after the course of study treatment.

The investigator will allow delegates of the insurance company to have access to the source data/documents as necessary to clarify a case of damage related to study participation. All involved parties will keep the patient data strictly confidential. A copy of the insurance certificate will be placed in the Investigator’s Site File.

16 STUDY REGISTRATION

The study is registered in the international trial registry ClinicalTrials.gov NCT02076373 (clinicaltrials.gov).

PUBLICATION POLICY

The trial protocol will be published e.g. in the journal ‘Trials’ after ethical approval by the PI Sven Wellmann, the co-investigator Christoph Rüegger, the Sponsor Hans-Ulrich Bucher, the trial mentor Christoph Bührer, the study nurse Brigitte Koller, and the specialist involved in MRI analyses, Cornelia Hagmann.

After the statistical analysis of this trial the sponsor will make every endeavour to publish the data in a medical journal.

The list of authors will include in all publications based on this trial data the local PI and one or two treating physicians of each contributing centre (depending on the number of recruited infants and the individual requirements of medical journals). Of each satellite centres, as described in chapter 10.7 and SOP-08 “Verlegung”, if at least one study patient has been cared the local PI will be included in all publications based on this trial data.

The order is defined as follows: First, second, and last position are reserved for study PI, the co-investigator, and the sponsor (Wellmann, Rüegger, and Bucher, respectively), 2 investigators of each centre in order of number of infants included in the study.

It is planned to publish the secondary outcomes e.g. on cUS findings and on MRI separately and before the primary outcome is achieved. According to the individual contribution of specialists, especially of Cornelia Hagmann regarding cUS and MRI, the first, second, or last position will be taken by the specialist.

With regard to publications, the principles of data protection must be observed for patient data as well as for data pertaining to participating physicians.

17 SIGNATURES

Sponsor:

This clinical trial protocol was subject to critical review and has been approved by the Sponsor and the Principial Investigator. The information herein is consistent with
- the current risk/benefit evaluation of the investigational product(s)
- the moral, ethical and scientific principles governing clinical research as set out in the current version of the Declaration of Helsinki, Good Clinical Practice and the respective SAMW guidelines.

Place/Date	Signature
Prof. Dr. med. Hans Ulrich Bucher	Klinik für Neonatologie, UniversitätsSpital Zürich

Coordinating Principal Investigator:

Place/Date	Signature
Prof. Dr. med. Sven Wellmann	Universitäts-Kinderspital beider Basel (UKBB)

Responsible Staff:

Place/Date Signature

Co-investigator, Dr. med. Christoph Rüegger, Klinik für Neonatologie USZ

Place/Date Signature

Study nurse, Claudia Knöpfli-Lenzin, Klinik für Neonatologie USZ

Place/Date	Signature
**Biometrician,** Dr. Beate Sick,	Universität Zürich

Place/Date Signature

Finances, Ms Iris Suter, Klinik für Neonatologie USZ

Place/Date Signature

Analysis of MRI, PD Dr. med. Cornelia Hagmann, Universitäts-Kinderspital Zürich,

Local Investigators

Signature

Place/Date

Aarau, KD Dr. med. Philipp Meyer Schiffer, Chefarzt

Signature

Place/Date

Basel, Prof. Dr. med. Sven Schulzke, Leitender Arzt

Signature

Place/Date

Bern, PD Dr. med. Mathias Nelle, Leitender Arzt

Signature

Place/Date

Chur, Dr. med. Brigitte Scharrer, Leitende Ärztin

Signature

Place/Date

Lausanne, Prof. Dr. med. Anita Truttmann, Leitende Ärztin

Signature

Place/Date

St. Gallen, Dr. med. Bjarte Rogdo, Leitender Arzt

Signature

Place/Date

Zürich, Prof. Dr. med. Jean-Claude Fauchère, Leitender Arzt

Signature

Place/Date

Berlin, Prof. Dr. med. Christoph Bührer, Klinikdirektor

Signature

Place/Date

Wien, Prof. Dr. med. Katrin Klebermass-Schrehof, Leitende Ärztin

Local Investigators from satellite centres

Signature
Place/Date

Bellinzona, Prof. Dr. med. Giacomo Simonetti

Signature
Place/Date

Biel, Dr. med. Mathias Gebauer

Signature
Place/Date

Fribourg, Dr. med. Benedikt Huber

Signature
Place/Date

Zürich, Stadtspital Triemli, Prof. Dr. med. Maren Tomaske

Signature
Place/Date

Zürich, Kinderspital Zürich, PD Dr. med. Vincenzo Cannizzaro

Signature
Place/Date

Zürich, Spital Zollikerberg, Dr. med. Marion Mönkhoff

Signature
Place/Date

Winterthur, Kantonsspital Winterthur, Dr. med. Michael Kleber

REFERENCES

1. Marlow N, Wolke D, Bracewell MA, Samara M. Neurologic and developmental disability at six years of age after extremely preterm birth. N Engl J Med 2005;352:9-19.
2. Larroque B, Ancel PY, Marret S, et al. Neurodevelopmental disabilities and special care of 5-year-old children born before 33 weeks of gestation (the EPIPAGE study): a longitudinal cohort study. Lancet 2008;371:813-20.
3. Ment LR, Allan WC, Makuch RW, Vohr B. Grade 3 to 4 intraventricular haemorrhage and Bayley scores predict outcome. Pediatrics 2005;116:1597-8; author reply 8.
4. Vohr BR, Wright LL, Poole WK, McDonald SA. Neurodevelopmental outcomes of extremely low birth weight infants <32 weeks' gestation between 1993 and 1998. Pediatrics 2005;116:635-43.
5. Luu TM, Ment LR, Schneider KC, Katz KH, Allan WC, Vohr BR. Lasting effects of preterm birth and neonatal brain haemorrhage at 12 years of age. Pediatrics 2009;123:1037-44.
6. Mercier CE, Dunn MS, Ferrelli KR, Howard DB, Soll RF. Neurodevelopmental outcome of extremely low birth weight infants from the Vermont Oxford network: 1998-2003. Neonatology 2009;97:329-38.
7. de Vries LS, van Haastert IC, Benders MJ, Groenendaal F. Myth: cerebral palsy cannot be predicted by neonatal brain imaging. Semin Fetal Neonatal Med 2011;16:279-87.
8. Adams-Chapman I, Hansen NI, Stoll BJ, Higgins R. Neurodevelopmental outcome of extremely low birth weight infants with posthaemorrhagic hydrocephalus requiring shunt insertion. Pediatrics 2008;121:e1167-77.
9. Vasileiadis GT, Gelman N, Han VK, et al. Uncomplicated intraventricular haemorrhage is followed by reduced cortical volume at near-term age. Pediatrics 2004;114:e367-72.
10. Patra K, Wilson-Costello D, Taylor HG, Mercuri-Minich N, Hack M. Grades I-II intraventricular haemorrhage in extremely low birth weight infants: effects on neurodevelopment. J Pediatr 2006;149:169-73.
11. Limperopoulos C, Soul JS, Haidar H, et al. Impaired trophic interactions between the cerebellum and the cerebrum among preterm infants. Pediatrics 2005;116:844-50.
12. Srinivasan L, Allsop J, Counsell SJ, Boardman JP, Edwards AD, Rutherford M. Smaller cerebellar volumes in very preterm infants at term-equivalent age are associated with the presence of supratentorial lesions. AJNR Am J Neuroradiol 2006;27:573-9.
13. Limperopoulos C, Bassan H, Gauvreau K, et al. Does cerebellar injury in premature infants contribute to the high prevalence of long-term cognitive, learning, and behavioral disability in survivors? Pediatrics 2007;120:584-93.
14. Felderhoff-Mueser U, Buhrer C. Clinical measures to preserve cerebral integrity in preterm infants. Early Hum Dev 2005;81:237-44.
15. Hamrick SE, Miller SP, Leonard C, et al. Trends in severe brain injury and neurodevelopmental outcome in premature newborn infants: the role of cystic periventricular leukomalacia. J Pediatr 2004;145:593-9.
16. Wilson-Costello D, Friedman H, Minich N, et al. Improved neurodevelopmental outcomes for extremely low birth weight infants in 2000-2002. Pediatrics 2007;119:37-45.
17. Groenendaal F, Termote JU, van der Heide-Jalving M, van Haastert IC, de Vries LS. Complications affecting preterm neonates from 1991 to 2006: what have we gained? Acta Paediatr 2010;99:354-8.
18. Whitelaw A, Jary S, Kmita G, et al. Randomized trial of drainage, irrigation and fibrinolytic therapy for premature infants with posthaemorrhagic ventricular dilatation: developmental outcome at 2 years. Pediatrics 2010;125:e852-8.
19. Volpe JJ. Brain injury in premature infants: a complex amalgam of destructive and developmental disturbances. Lancet Neurol 2009;8:110-24.

20. Himpens E, Oostra A, Franki I, Van Maele G, Vanhaesebrouck P, Van den Broeck C. Predictability of cerebral palsy and its characteristics through neonatal cranial ultrasound in a high-risk NICU population. Eur J Pediatr 2010;169:1213-9.
21. De Vries LS, Van Haastert IL, Rademaker KJ, Koopman C, Groenendaal F. Ultrasound abnormalities preceding cerebral palsy in high-risk preterm infants. J Pediatr 2004;144:815-20.
22. Fauchere JC, Dame C, Vonthein R, et al. An approach to using recombinant erythropoietin for neuroprotection in very preterm infants. Pediatrics 2008;122:375-82.
23. Dzietko M, Felderhoff-Mueser U, Sifringer M, et al. Erythropoietin protects the developing brain against N-methyl-D-aspartate receptor antagonist neurotoxicity. Neurobiol Dis 2004;15:177-87.
24. Kellert BA, McPherson RJ, Juul SE. A comparison of high-dose recombinant erythropoietin treatment regimens in brain-injured neonatal rats. Pediatr Res 2007;61:451-5.
25. Iwai M, Cao G, Yin W, Stetler RA, Liu J, Chen J. Erythropoietin promotes neuronal replacement through revascularization and neurogenesis after neonatal hypoxia/ischemia in rats. Stroke 2007;38:2795-803.
26. Zacharias R, Schmidt M, Kny J, et al. Dose-dependent effects of erythropoietin in propofol anesthetized neonatal rats. Brain Res 2010;1343:14-9.
27. Minnerup J, Heidrich J, Rogalewski A, Schabitz WR, Wellmann J. The efficacy of erythropoietin and its analogues in animal stroke models: a meta-analysis. Stroke 2009;40:3113-20.
28. Jerndal M, Forsberg K, Sena ES, et al. A systematic review and meta-analysis of erythropoietin in experimental stroke. J Cereb Blood Flow Metab 2010;30:961-8.
29. Widness JA, Veng-Pedersen P, Modi NB, Schmidt RL, Chestnut DH. Developmental differences in erythropoietin pharmacokinetics: increased clearance and distribution in fetal and neonatal sheep. J Pharmacol Exp Ther 1992;261:977-84.
30. Brines ML, Ghezzi P, Keenan S, et al. Erythropoietin crosses the blood-brain barrier to protect against experimental brain injury. Proc Natl Acad Sci U S A 2000;97:10526-31.
31. Juul SE, McPherson RJ, Farrell FX, Jolliffe L, Ness DJ, Gleason CA. Erytropoietin concentrations in cerebrospinal fluid of nonhuman primates and fetal sheep following high-dose recombinant erythropoietin. Biol Neonate 2004;85:138-44.
32. Ehrenreich H, Weissenborn K, Prange H, et al. Recombinant human erythropoietin in the treatment of acute ischemic stroke. Stroke 2009;40:e647-56.
33. Ehrenreich H, Hinze-Selch D, Stawicki S, et al. Improvement of cognitive functions in chronic schizophrenic patients by recombinant human erythropoietin. Mol Psychiatry 2007;12:206-20.
34. Wustenberg T, Begemann M, Bartels C, et al. Recombinant human erythropoietin delays loss of gray matter in chronic schizophrenia. Mol Psychiatry 2011;16:26-36, 1.
35. Zhu C, Kang W, Xu F, et al. Erythropoietin improved neurologic outcomes in newborns with hypoxic-ischemic encephalopathy. Pediatrics 2009;124:e218-26.
36. Elmahdy H, El-Mashad AR, El-Bahrawy H, El-Gohary T, El-Barbary A, Aly H. Human recombinant erythropoietin in asphyxia neonatorum: pilot trial. Pediatrics 2010;125:e1135-42.
37. Ohlsson A, Aher SM. Early erythropoietin for preventing red blood cell transfusion in preterm and/or low birth weight infants. Cochrane Database Syst Rev 2006;3:CD004863.
38. Aher SM, Ohlsson A. Early versus late erythropoietin for preventing red blood cell transfusion in preterm and/or low birth weight infants. Cochrane Database Syst Rev 2006;3:CD004865.
39. Aher S, Ohlsson A. Late erythropoietin for preventing red blood cell transfusion in preterm and/or low birth weight infants. Cochrane Database Syst Rev 2006;3:CD004868.
40. Bierer R, Peceny MC, Hartenberger CH, Ohls RK. Erythropoietin concentrations and neurodevelopmental outcome in preterm infants. Pediatrics 2006;118:e635-40.

41. Brown MS, Eichorst D, Lala-Black B, Gonzalez R. Higher cumulative doses of erythropoietin and developmental outcomes in preterm infants. Pediatrics 2009;124:e681-7.
42. Neubauer AP, Voss W, Wachtendorf M, Jungmann T. Erythropoietin improves neurodevelopmental outcome of extremely preterm infants. Ann Neurol 2010;67:657-66.
43. Juul SE, Stallings SA, Christensen RD. Erythropoietin in the cerebrospinal fluid of neonates who sustained CNS injury. Pediatr Res 1999;46:543-7.
44. Ehrenreich H, Hasselblatt M, Dembowski C, et al. Erythropoietin therapy for acute stroke is both safe and beneficial. Mol Med 2002;8:495-505.
45. Wang L, Zhang Z, Wang Y, Zhang R, Chopp M. Treatment of stroke with erythropoietin enhances neurogenesis and angiogenesis and improves neurological function in rats. Stroke 2004;35:1732-7.
46. Juul SE, McPherson RJ, Bauer LA, Ledbetter KJ, Gleason CA, Mayock DE. A phase I/II trial of high-dose erythropoietin in extremely low birth weight infants: pharmacokinetics and safety. Pediatrics 2008;122:383-91.
47. McAdams RM, McPherson RJ, Mayock DE, Juul SE. Outcomes of extremely low birth weight infants given early high-dose erythropoietin. J Perinatol 2012.
48. Brown MS, Jones MA, Ohls RK, Christensen RD. Single-dose pharmacokinetics of recombinant human erythropoietin in preterm infants after intravenous and subcutaneous administration. J Pediatr 1993;122:655-7.
49. Ohls RK, Veerman MW, Christensen RD. Pharmacokinetics and effectiveness of recombinant erythropoietin administered to preterm infants by continuous infusion in total parenteral nutrition solution. J Pediatr 1996;128:518-23.
50. Leuchter H-V. Neuroprotective effects of early EPO in preterm infants: an MRI study Oral presentation at the annual meeting of the ESPR in Newcastle, UK 2011.
51. Mainie P. Is there a role for erythropoietin in neonatal medicine? Early Hum Dev 2008;84:525-32.
52. Von Kohorn I, Ehrenkranz RA. Anemia in the preterm infant: erythropoietin versus erythrocyte transfusion--it's not that simple. Clin Perinatol 2009;36:111-23.
53. Schneider JK, Gardner DK, Cordero L. Use of recombinant human erythropoietin and risk of severe retinopathy in extremely low-birth-weight infants. Pharmacotherapy 2008;28:1335-40.
54. Slusarski JD, McPherson RJ, Wallace GN, Juul SE. High-dose erythropoietin does not exacerbate retinopathy of prematurity in rats. Pediatr Res 2009;66:625-30.
55. Jelkmann W. Biosimilar epoetins and other "follow-on" biologics: update on the European experiences. Am J Hematol 2010;85:771-80.
56. Maier RF, Obladen M, Muller-Hansen I, et al. Early treatment with erythropoietin beta ameliorates anemia and reduces transfusion requirements in infants with birth weights below 1000 g. J Pediatr 2002;141:8-15.
57. Melchers P, Preuß U. Kaufman Assessment Battery for Children (deutsche Version): Pearson Assessment; 2009.
58. Tellegen PJ, Laros JA, Petermann F. Snijders-Oomen non-verbaler Intelligenztest von 2,5 bis 7 Jahre (SON-R 2,5-7), Testbatterie zur sprachfreien Messung der allgemeinen Intelligenz: Hogrefe; 2007.
59. Hack M, Taylor HG, Drotar D, et al. Poor predictive validity of the Bayley Scales of Infant Development for cognitive function of extremely low birth weight children at school age. Pediatrics 2005;116:333-41.
60. Leversen KT, Sommerfelt K, Elgen IB, et al. Prediction of outcome at 5 years from assessments at 2 years among extremely preterm children. Acta Paediatr 2011.

61. Johnson S, Hennessy E, Smith R, Trikic R, Wolke D, Marlow N. Academic attainment and special educational needs in extremely preterm children at 11 years of age: the EPICure study. Arch Dis Child Fetal Neonatal Ed 2009;94:F283-9.
62. Largo RH, Graf S, Kundu S, Hunziker U, Molinari L. Predicting developmental outcome at school age from infant tests of normal, at-risk and retarded infants. Dev Med Child Neurol 1990;32:30-45.
63. An international classification of retinopathy of prematurity. II. The classification of retinal detachment. The International Committee for the Classification of the Late Stages of Retinopathy of Prematurity. Arch Ophthalmol 1987;105:906-12.
64. Parry G, Tucker J, Tarnow-Mordi W. CRIB II: an update of the clinical risk index for babies score. Lancet 2003;361:1789-91.

University of Zurich[UZH]

Epidemiology, Biostatistics
and Prevention Institute,
Biostatistics Department
+41 44 634 46 41
www.biostat.uzh.ch

EpoRepair

Statistical Analysis Plan for Prof. Dr. med. Sven Wellmann

Dr. Stefanie von Felten (*stefanie.vonfelten@uzh.ch*)

Version 2.0 of June 13, 2019

DISCLAIMER

This report was written by the Research Methods Consulting Unit of the University of Zurich, Epidemiology, Biostatistics and Prevention Institute, Biostatistics Department (EBPI). Any citation of results or comments (or parts thereof) given in this report in any format (including: paper, presentation, abstract, grant application) or the naming of EBPI or persons of the EBPI requires the explicit approval of the senior statistician (Dr. Stefanie von Felten). This is especially true for citations in the "acknowledgments".

Contents

1 Background **2**

2 Outcomes **2**
2.1 Primary outcome . . . 2
2.2 Secondary outcomes . . . 2
2.2.1 Secondary outcomes at 5 years (like primary outcome) . . . 2
2.2.2 Secondary outcomes at 2 years . . . 2
2.2.3 Secondary outcomes up to term equivalent age . . . 3
2.2.4 Other outcomes . . . 4

3 Hypothesis **4**

4 Analysis data set **4**

5 Planned analyses **5**
5.1 Primary analysis . . . 5
5.2 Secondary analyses . . . 6
5.2.1 Subgroup analyses for the primary outcome . . . 6
5.2.2 Analysis of secondary outcomes . . . 6
5.3 Descriptive analyses . . . 7

6 Interim analysis **7**

7 Safety analysis **7**

8 Handling of missing data and drop-outs **7**

9 Deviations from the original statistical plan 8

10 References 8

1 Background

EpoRepair is a randomized, double-blind, placebo-controlled, international multi-center study to investigate the effect of intravenously administered Recormon (recombinant erythropoietin, hereafter referred to as EPO) on the outcome of very preterm infants (born before 32 gestational weeks and/or 1500 g of birth weight) after intraventricular haemorrhage (grade II-IV, diagnosed by cranial ultrasound at a chronological age < 9 d). The aim is to evaluate whether EPO has the potential to ameliorate the neurodevelopmental sequelae of intraventricular haemorrhage. EPO as a verum is compared to NaCl 0.9 % as a placebo. Randomization was done 1:1, blockwise within each center (using a separate randomization list for each of the nine participating centers). The trial protocol was published by Rüegger et al. (2015).

2 Outcomes

2.1 Primary outcome

The primary efficacy outcome of this trial ist the intelligence quotient assessed by the Kaufman Assessment Battery for Children (Kaufman-ABC, German version) at 5 years of age. Children with speaking problems at 5 years will be assessed by an alternative, non-verbal intelligence test, the Snijders-Oomen non-verbal intelligence test (SON-R 2.5-7, German version), and the result will be used instead of Kaufman-ABC.

2.2 Secondary outcomes

Secondary outcomes are grouped by the follow-up time at/up to which they are measured.

2.2.1 Secondary outcomes at 5 years (like primary outcome)

- Normal overall developmental outcome (binary). Normal development at 5 years is defined as the combination of normal neurological evaluation, IQ > 84, and no neurodevelopmental deficits (all three conditions must be met).
- Mortality (binary)
- Developmental outcome categorized as normal, non-normal or dead (multinomial or binary: normal vs. non-normal or dead), combining the two outcomes just above into one[1]

2.2.2 Secondary outcomes at 2 years

- Bayley Scales III of Infant Development (BSID-III), combined score (continuous outcome, norm value 100, range ca. 30–150)

[1] This outcome was neither prespecified in the study protocol nor in the published study protocol. It was added to take into account the considerable proportion of children who die before the age of 5 years.

  - Subscore for motion (continuous, norm value 100)
  - Subscore for cognition (continuous, norm value 100)

- Presence of impairment of motor function (cerebral palsy, binary)
- Presence of impairment of neurosensory function (blindness or deafness, binary)
- Mortality (binary)

2.2.3 Secondary outcomes up to term equivalent age

These outcomes are measured at slightly different times, some at 40 weeks postmenstrual age (PMA), some at 36 weeks PMA and some at 28 days of life (DOL).

Outcomes from advanced MRI at 40 weeks PMA

- Global brain abnormality score according to Kidokoro et al. (2013) (continuous from 0–40 and binary normal/mild vs. moderate/severe)[2]
  - Subscore for white matter abnormality scale (continuous from 0-17 and binary: normal/mild vs moderate/severe)
  - Subscore for cortical grey matter abnormality scale (0-9), binary: normal/mild vs moderate/ severe)
  - Subscore for deep grey matter abnormality scale (0-7), binary: normal/mild vs moderate/ severe)
  - Subscore for cerebellar abnormality scale (0-7), binary: normal/mild vs moderate/ severe)

Outcomes from cerebral ultrasound (cUS) until discharge (28 DOL and 36 weeks PMA)

- Brain injury grading (normal, moderately abnormal, severe brain injury, binary as normal/moderate vs severe, or normal vs moderate/severe)
- Posthaemorrhagic hydrocephalus (Grade 0–3, binary as ≤ 1 vs > 1)
- Composite cUS score at 36 weeks PMA, according to Skiöld et al. (2019) (continuous from 0–55 and binary: normal/mild vs moderate/severe)[3]

More outcomes up to term equivalent age

- Mortality (binary outcome)
- White matter disease (periventricular leucomalacia, binary)[4]
- Septicaemia (binary)[4]

[2] Brain abnormality scores from Kidokoro et al. (2013) will be used instead of the brain maturation scores described by Leuchter et al. (2014).

[3] This outcome will be assessed for all recruited children in only one center, as opposed to the other cUS outcomes that were assessed in each center. An amendment of the study protocol is planned for this new secondary outcome.

[4] These secondary outcomes are mentioned in the study protocol from Zürich, Version 1.11 from March 20, 2017, but not in the protocol paper by Rüegger et al. (2015)

- Necrotising enterocolitis or focal gastrointestinal perforation (FIP) (binary)[4]
- Bronchopulmonary dysplasia (oxygen dependency at 36 weeks PMA, binary)[4]
- Retinopathy (binary)[4]
- Length of stay in hospital (days)[4]

2.2.4 Other outcomes

The outcomes listed here (measured at 28 DOL and/or 36 weeks PMA) will only be analyzed descriptively:

- Severity of anaemia
  - Hematocrit (%)
  - Hemoglobin (g/dl)
  - Number of transfusions
- Weight (g)
- Head circumference (cm)
- Length of supplemental O_2 (PMA at last day with supplemental O_2)
- Total number of days with supplemental O_2

3 Hypothesis

The alternative hypothesis for the primary endpoint is that EPO improves the intelligence quotient at 5 years of age compared to Placebo. The null hypothesis is no improvement with regard to the primary endpoint by EPO compared to Placebo.

Although the main study hypothesis is one-sided, we will apply two-sided tests. Estimates and confidence intervals will provide information about the direction of potential differences between the EPO and the Placebo arm.

4 Analysis data set

The **full analysis set (FAS)** will contain all children randomized to the trial whose parents gave informed consent, except one child who had to be excluded from the study after randomization due to severe genetic damage which was an exclusion criterion (another child was recruited instead).

Due to missing outcome measurements (and maybe explanatory variables) we will use multiple imputation to be able to analyze the complete FAS (see also Sections 5.1 and 8). In addition, we will do complete case analyses and optionally apply a special method to account for missing data due to death (sensitivity analysis S4 in Section 5.1).

5 Planned analyses

The statistical analysis will be performed in three stages, including (1) secondary outcomes measured up to term equivalent age, (2) secondary outcomes measured after 2 years follow-up and (3) the primary outcome and secondary outcomes measured after 5 years follow-up.

All statistical analyses will be performed using the Statistical software environment R (R Core Team, 2018). We will always report estimates and 95 % confidence intervals (corresponding to a significance level of 0.05) together with p-values. Information on specific R packages is given below. Due to the large number of secondary outcomes, we will adjust p-values for the comparison of secondary outcomes among treatment arms for multiplicity, using the Bonferroni-Holm method (Holm, 1979) and report them alongside the unadjusted p-values.

5.1 Primary analysis

The primary outcome, IQ measured by Kaufman-ABC (or SON-R), will be compared between treatment arms (EPO vs. Placebo) using a linear mixed-effects model with "treatment" as fixed explanatory factor and a random intercept per "center". This will result in an estimate (and 95 % CI) of the EPO treatment effect on IQ. The model assumptions will be checked by visual assessment of the residuals and log-transformation of the primary outcome may be considered in case of violations. We will use the R package lme4 (Bates et al., 2015) to fit the models and the R package lmerTest to derive p-values via the Satterthwaite approximation (Kuznetsova et al., 2017).

It has to be noted, that a comparison of IQ among survivors who performed an IQ test is prone to bias due to the large proportion of children who die before the age of 5 years and due to other losses to follow-up (20 % loss was expected in total, ca. 15 % of the newborn babies already died in hospital). The bias would be particularly high if the reasons for, or the frequency of, losses differed between trial arms (see e.g. Altman, 2009). Adhering to the method proposed in the protocol and its published version (Rüegger et al., 2015), we will use multiple imputation for the missing IQ values (van Buuren and Groothuis-Oudshoorn, 2011). We thereby assume that data are missing at random (MAR) and we treat missingness due to death in the same way as missingness due to other reasons. For more details see Section 8. The analysis will be performed on the FAS, in line with the intention-to-treat (ITT) principle.

The following sensitivity analyses will be done in addition to the "main primary analysis" just described:

S1 Covariate adjusted analysis with birth weight of the child, severity of the intraventricular hemorrhage (Grade II, III or IV), steroid administration before birth, steroid administration after birth and Socio-Economic Score (SES) of the mother as additional explanatory variables compared to the main primary analysis.

S2 Model described for the main primary analysis but performed on the complete cases in the FAS only (without multiple imputation, not in line with ITT)

S3 Optional: model described for the main primary analysis but performing a modified version of multiple imputation, using the random indicator method (Jolani, 2012) to account for "non-ignorable missing data" (to handle the problem that children for whom IQ data are missing may differ from those with IQ data even after accounting for the observed information).

S4 Optional: the method proposed by Hayden et al. (2005), which specifically deals with missing data due to death (as opposed to missing data in patients that are alive), will be applied. This

method of principal stratification will estimate a "survivor average causal effect" (SACE), a concept introduced by (Rubin, 2000). The SACE would be the effect of Epo on those children who would have survived under both treatments (Epo and Placebo).

5.2 Secondary analyses

5.2.1 Subgroup analyses for the primary outcome

The following subgroups will be examined:

- Center (one level for each center, or at least for those recruiting $\geq$ 10 children)
- Hydrocephalus at cUS before 8 DOL (Grade ≤ 1 vs. > 1)
- Severity of intraventricular haemorrhage, IVH, at cUS before 8 DOL (with infarction vs. without infarction)

We will assess whether the treatment effect of EPO differs among subgroups of patients by adding the subgroup factor and the subgroup $\times$ treatment interaction to the statistical model described for the main primary analysis above (Section 5.1), except that for the subgroup "center" we will drop the random intercept per center. A significant subgroup $\times$ treatment interaction will indicate that the treatment effect differs between subgroups (or in at least one level of the subgroup factor in case of > 2 levels).

We will use the FAS with multiply imputed missing values for these subgroup analyses.

5.2.2 Analysis of secondary outcomes

Continuous secondary outcomes will be compared between treatment arms (EPO vs. Placebo) using a linear mixed-effects model with "treatment" as fixed explanatory factor and a random intercept per "center", like the primary outcome. We will report the resulting estimates of the EPO treatment effects together with 95 % confidence intervals and p-values (unadjusted and adjusted for multiple testing). The model assumptions will be checked by visual assessment of the residuals and either log-transformation of the outcome or the use of a generalized linear mixed-effects model (e.g. with Poisson error) may be considered in case of violations. For the outcome BSID-III at two years (combined score and subscores) we will add a sensitivity analysis to adjust for additional covariates, as specified as sensitivity analysis S1 for the primary outcome (Section 5.1). Moreover, the methods described for sensitivity analyses S3 and S4 may also be applied to continuous secondary outcomes.

Binary secondary outcomes will be compared between treatment arms (EPO vs. Placebo) using a generalized linear mixed-effects model with binomial error (referred to as logistic regression in the study protocol), again with "treatment" as fixed explanatory factor and a random intercept per "center". The resulting odds ratio (OR) estimates of the EPO treatment effect will be reported together with 95 % confidence intervals and p-values. Binary secondary outcomes from cUS (severe brain injury, posthaemorrhagic hydrocephalus grade > 1) were measured three times: at baseline, 28 DOL and 36 weeks PMA. The measurement taken at baseline and the time of outcome measurement (28 DOL vs. 36 weeks PMA) will be used as additional fixed explanatory variables in the model. A random intercept per patient (nested in center) will be added to the model, to account for the non-independence of the repeated outcome measurements. In addition, we will assess the treatment $\times$ time interaction. We will use the R package lme4 (Bates et al., 2015) to fit the models for binary outcomes and derive p-values using likelihood ratio tests.

Developmental outcome (normal, non-normal or dead) will be compared between treatment arms by a chi-squared test and will also be analyzed as binary outcome (normal vs. non-normal or dead) as described above.

The length of stay (LOS) in hospital will be analyzed as time to hospital discharge, using a competing risks model to account for the competing risk of in-hospital death (as recommended in Brock et al., 2011). We will use the R package survival (Terry M. Therneau and Patricia M. Grambsch, 2000) to fit a cause-specific proportional hazards model. For the sake of simplicity, we will include treatment and center as fixed effects (no random center effect).

We will once use the multiply imputed FAS and once the complete cases of the FAS for all secondary analyses.

5.3 Descriptive analyses

Relevant demographic and baseline characteristics, as well as all outcomes listed in Section 2 will be summarized per trial arm for each of the analysis sets. Mean and standard deviation (or if more appropriate, median and interquartile range) will be reported for continuous variables. Frequencies and percentages will be reported for categorical variables.

In addition, outcomes will also be graphically displayed.

6 Interim analysis

An interim safety analysis for the Data Safety Monitoring Board (DSMB) was planned when the first 50 patients passed visit 2, and was already performed in 2016.

7 Safety analysis

We will descriptively analyze AEs and SAEs, including relation to the investigational medical product (IMP) up to 40 weeks PMA. We will summarize frequencies and percentages by trial arm and also show the full list of AEs with description.

8 Handling of missing data and drop-outs

We will multiply impute missing outcome and covariate data due to death or loss to follow-up in the FAS. Multiple imputation will be performed by the R package mice (van Buuren and Groothuis-Oudshoorn, 2011), and will be applied seperately at each stage of analysis (see Section 5). The imputation model will use all relevant baseline variables, the randomized treatment and outcome variables collected up to the time of analysis, potentially using correlations among outcomes for imputation in case the amount of missing data differs among outcomes. We will produce m=20 imputations per missing value and analyze the 20 resulting complete data sets using Rubin's rules to pool the results.

While multiple imputation assumes missingness at random (MAR), missingness not at random can be investigated using the random indicator method proposed by Jolani (2012), mentioned also in van Buuren (2018). The latter will be used for sensitivity analysis S3.

In addition, a principal stratification approach suggested by Hayden et al. (2005) may be applied in order to estimate the "survivor average causal effect" (SACE), as proposed for sensitivity analysis S4.

9 Deviations from the original statistical plan

The statistical analyses proposed in this document are generally in line with those proposed in the protocol and its published version (Rüegger et al., 2015), but include a few slight modifications or additions:

- Brain abnormality scores from Kidokoro et al. (2013) will be used as secondary outcomes instead of the brain maturation scores described by Leuchter et al. (2014) (see footnote[2] above).
- Composite cUS scores will be centrally assessed, in addition to the cUS outcomes that are assessed in each center (see footnote[3] above).
- Some outcomes were only listed in the study protocol (Zürich, Version 1.11 of March 20, 2017) but not in the published version (Rüegger et al., 2015) (see footnote[4] above).
- "Other outcomes" listed in Section 2.2.4 were not listed anywhere before and are therefore only analyzed descriptively
- Due to the large number of centers compared to the number of patients we use a random intercept per center instead of modelling center as fixed effect. This allows to include additional covariates, as specified in sensitivity analysis S1.
- The sensitivity analysis S3 was added to assess the assumption of missingness at random (MAR) and S4 was added because missingness of data due to death is different from missingness that arises when a patient still alive is not measured.

These deviations and all deviations (if any) from the statistical analysis plan outlined in this document will be listed in the statistical analysis report.

10 References

Altman, D. G. (2009). Missing outcomes in randomized trials: addressing the dilemma. *Open Medicine* **3** e51.

Bates, D., Mächler, M., Bolker, B. and Walker, S. (2015). Fitting linear mixed-effects models using lme4. *Journal of Statistical Software, Articles* **67** 1–48.
URL https://www.jstatsoft.org/v067/i01

Brock, G. N., Barnes, C., Ramirez, J. A. and Myers, J. (2011). How to handle mortality when investigating length of hospital stay and time to clinical stability. *BMC medical research methodology* **11** 144.

Hayden, D., Pauler, D. K. and Schoenfeld, D. (2005). An estimator for treatment comparisons among survivors in randomized trials. *Biometrics* **61** 305–310.

Holm, S. (1979). A simple sequentially rejective multiple test procedure. *Scandinavian Journal of Statistics* **6** 65–70.
URL http://www.jstor.org/stable/4615733

Jolani, S. (2012). *Dual imputation strategies for analyzing incomplete data*. Ph.D. thesis, Utrecht University.

Kidokoro, H., Neil, J. and Inder, T. (2013). New mr imaging assessment tool to define brain abnormalities in very preterm infants at term. *American Journal of Neuroradiology* **34** 2208–2214.

Kuznetsova, A., Brockhoff, P. and Christensen, R. (2017). lmertest package: Tests in linear mixed effects models. *Journal of Statistical Software, Articles* **82** 1–26.
URL https://www.jstatsoft.org/v082/i13

Leuchter, R. H.-V., Gui, L., Poncet, A., Hagmann, C., Lodygensky, G. A., Martin, E., Koller, B., Darqué, A., Bucher, H. U. and Hüppi, P. S. (2014). Association between early administration of high-dose erythropoietin in preterm infants and brain mri abnormality at term-equivalent age. *Jama* **312** 817–824.

R Core Team (2018). *R: A Language and Environment for Statistical Computing*. R Foundation for Statistical Computing, Vienna, Austria.
URL https://www.R-project.org/

Rubin, D. (2000). Discussion of causal inference without counterfactuals. *Journal of the American Statistical Association* **95** 435–438.

Rüegger, C. M., Hagmann, C. F., Bührer, C., Held, L., Bucher, H. U., Wellmann, S., Investigators, E. et al. (2015). Erythropoietin for the repair of cerebral injury in very preterm infants (eporepair). *Neonatology* **108** 198–204.

Skiöld, B., Hallberg, B., Vollmer, B., Ådén, U., Blennow, M. and Horsch, S. (2019). A novel scoring system for term-equivalent-age cranial ultrasound in extremely preterm infants. *Ultrasound in medicine & biology* **45** 786–794.

Terry M. Therneau and Patricia M. Grambsch (2000). *Modeling Survival Data: Extending the Cox Model*. Springer, New York.

van Buuren, S. (2018). *Flexible imputation of missing data*. Chapman and Hall/CRC.

van Buuren, S. and Groothuis-Oudshoorn, K. (2011). mice: Multivariate imputation by chained equations in R. *Journal of Statistical Software* **45** 1–67.
URL http://www.jstatsoft.org/v45/i03/

This document was generated on Juni 13, 2019 at 09:17.
