# Supplementary material for: Safety and Short-term Outcomes of High-Dose Erythropoietin in Preterm Infants With Intraventricular Hemorrhage: The EpoRepair Randomized Clinical Trial
Source: JAMA Netw Open. 2022 Dec 2;5(12):e2244744. doi: 10.1001/jamanetworkopen.2022.44744 (PMC9719050; doi:10.1001/jamanetworkopen.2022.44744)
Supplement: Supplement 2. — eFigure 1. Kaplan-Meier Curves for Overall Survival After Birth in the Erythropoietin (Epo) and Placebo Group in Regard to Days of Life eFigure 2. Kaplan-Meier Curves for Overall Survival After Birth in the Erythropoietin (Epo) and Placebo Group in Regard to Postmenstrual Age (Weeks) eTable. Secondary Outcomes Up to Term Equivalent Age or Hospital Discharge eFigure 3. Empirical Cumulative Distribution of Global Brain Abnormality Scores in the Erythropoietin (Epo) and Placebo Group eFigure 4. Forest Plot of Odds Ratio (OR) Estimates (Boxes) and 95% Confidence Intervals (Lines) of the Erythropoietin (Epo) vs Placebo Treatment Effect on All Binary Secondary Outcomes Shown in the eTable of the Supplement File [file jamanetwopen-e2244744-s002.pdf]

Supplemental Online Content

Wellmann S, Hagmann CF, von Felten S, et al; Erythropoietin for the Repair of Cerebral Injury in Very Preterm Infants (EpoRepair) Investigators. Safety and short-term outcomes of high-dose erythropoietin in preterm infants with intraventricular hemorrhage: the EpoRepair randomized clinical trial. *JAMA Netw Open*. 2022;5(12):e2244744. doi:10.1001/jamanetworkopen.2022.44744

eFigure 1. Kaplan-Meier Curves for Overall Survival After Birth in the Erythropoietin (Epo) and Placebo Group in Regard to Days of Life

eFigure 2. Kaplan-Meier Curves for Overall Survival After Birth in the Erythropoietin (Epo) and Placebo Group in Regard to Postmenstrual Age (Weeks)

eTable. Secondary Outcomes Up to Term Equivalent Age or Hospital Discharge

eFigure 3. Empirical Cumulative Distribution of Global Brain Abnormality Scores in the Erythropoietin (Epo) and Placebo Group

eFigure 4. Forest Plot of Odds Ratio (OR) Estimates (Boxes) and 95% Confidence Intervals (Lines) of the Erythropoietin (Epo) vs Placebo Treatment Effect on All Binary Secondary Outcomes Shown in the eTable of the Supplement File

This supplemental material has been provided by the authors to give readers additional information about their work.

© 2022 Wellmann S et al. *JAMA Network Open.*

eFigure 1. Kaplan-Meier Curves for Overall Survival After Birth in the Erythropoietin (Epo) and Placebo Group in Regard to Days of Life

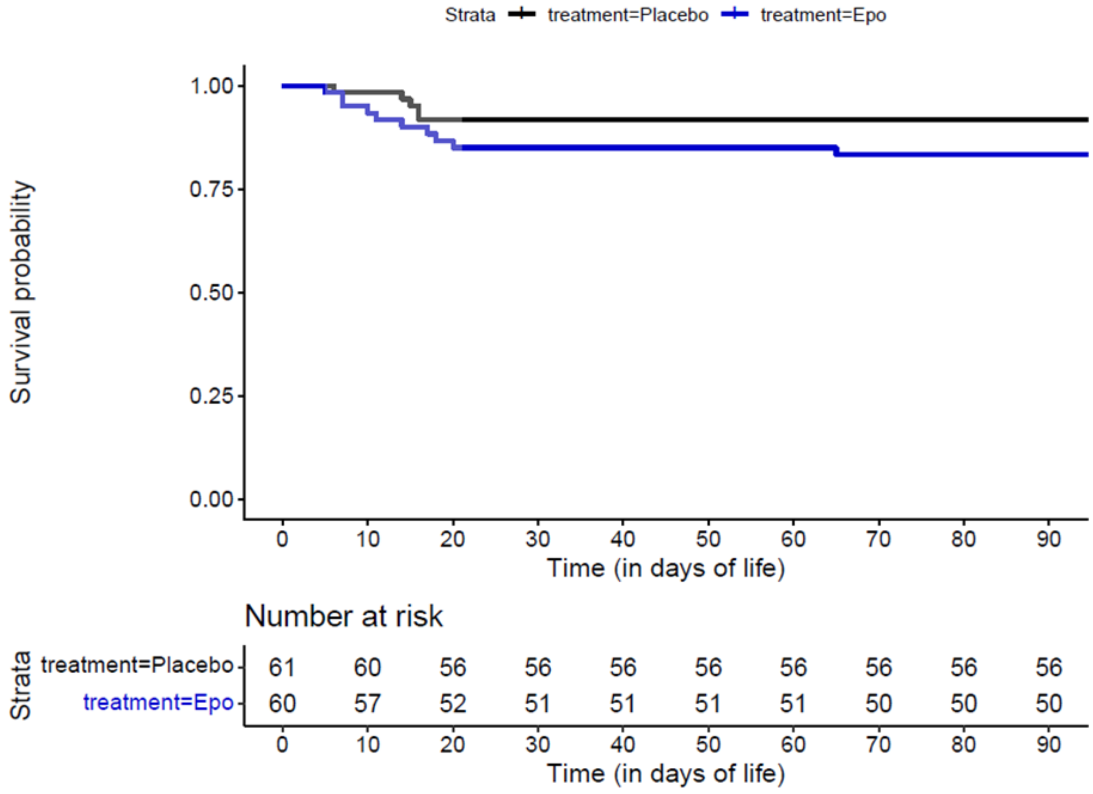

eFigure 2. Kaplan-Meier Curves for Overall Survival After Birth in the Erythropoietin (Epo) and Placebo Group in Regard to Postmenstrual Age (Weeks)

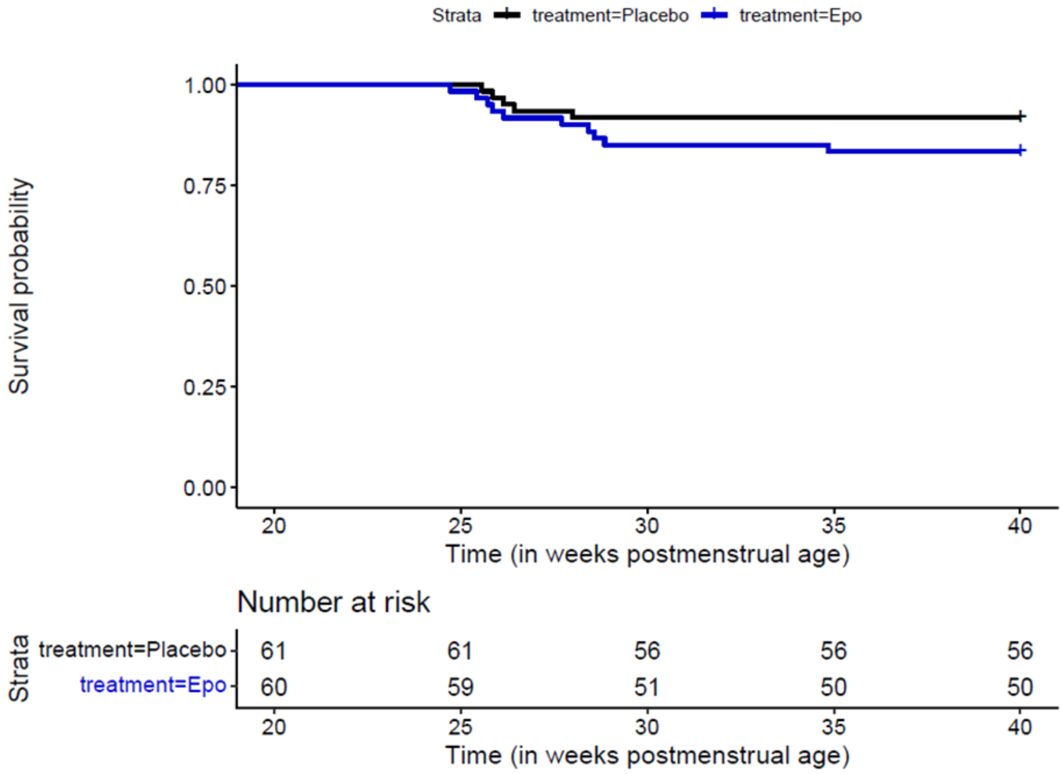

© 2022 Wellmann S et al. *JAMA Network Open.*

eTable. Secondary Outcomes Up to Term Equivalent Age or Hospital Discharge

**Outcome**	**Erythropoietin group[a] (n=60)**	**Placebo group[a] (n=61)**	**Effect size estimate (95% CI)[b]**	**p**
MRI outcome scores				
Global brain abnormality	8.5 [6.0– 12.3]	9.0 [7.0–11.3]	MD 0.65 (-1.79 to 3.15)	0.60
White matter abnormality	6.0 [3.8–8.0]	6.0 [4.0–7.0]	MD 0.59 (-0.64 to 1.81)	0.35
Moderate/severe cortical grey matter abnormality[c]	20 (42.6)	27 (52.9)	OR 0.66 (0.29 to 1.50)	0.32
Moderate/severe deep grey matter abnormality[c]	6 (12.5)	7 (13.5)	OR 0.93 (0.28 to 3.12)	0.91
Moderate/severe cerebellar abnormality[d]	19 (40.4)	22 (42.3)	OR 0.92 (0.40 to 2.06)	0.83
Clinical outcomes				
PHH with drainage DOL 28	11 (18.3)	9 (14.8)	OR 1.30 (0.49 to 3.48)	0.60
PHH with drainage PMA 36	11 (18.3)	8 (13.1)	OR 1.49 (0.56 to 4.13)	0.43
Culture-proven infection	14 (23.3)	15 (24.6)	OR 0.92 (0.39 to 2.14)	0.85
NEC or FIP	12 (20.0)	7 (11.5)	OR 1.93 (0.72 to 5.55)	0.20
Supplemental oxygen at PMA 36	18 (32.7)	26 (43.3)	OR 0.60 (0.26 to 1.33)	0.21
ROP 2 or higher	18 (30.0)	21 (34.4)	OR 0.91 (0.39 to 2.13)	0.83
PVL at PMA 36	14 (28.0)	12 (20.7)	OR 1.50 (0.62 to 3.72)	0.37
Death up to TEA	10 (16.7)	5 (8.2)	OR 2.24 (0.74 to 7.66)	0.15
Length of stay	91 (85 to 105)	85 (80 to 99)	HR 0.9 (0.6 to 1.35)	0.62

[a] Median and inter-quartile range are shown for the MRI outcomes global brain abnormality and white matter abnormality, frequencies and % for binary outcomes (all clinical outcomes) and the median length of stay in hospital estimated by the Kaplan-Meier method (with 95 % CI) for length of stay in hospital (analyzed as time to discharge alive).

[b] Mean difference (MD) for continuous MRI outcome scores, odds ratio (OR) for binary outcomes and hazard ratio (HR) for length of stay in hospital.

[c] These MRI outcomes were dichotomized as moderate/severe vs. normal/mild.

[d] PVL, periventricular leukomalacia: cystic or diffuse (duration >7 days).

Abbreviations: DOL: day of life, PHH: post-hemorrhagic hydrocephalus; NEC: necrotizing enterocolitis; FIP: focal intestinal perforation; ROP: retinopathy of prematurity; PVL: periventricular leukomalacia; PMA 36: 36 weeks post-menstrual age, TEA: term-equivalent age.

© 2022 Wellmann S et al. *JAMA Network Open.*

eFigure 3. Empirical Cumulative Distribution of Global Brain Abnormality Scores in the Erythropoietin (Epo) and Placebo Group. The blue and the black broken vertical lines represent the median for the erythropoietin and the placebo group, respectively.

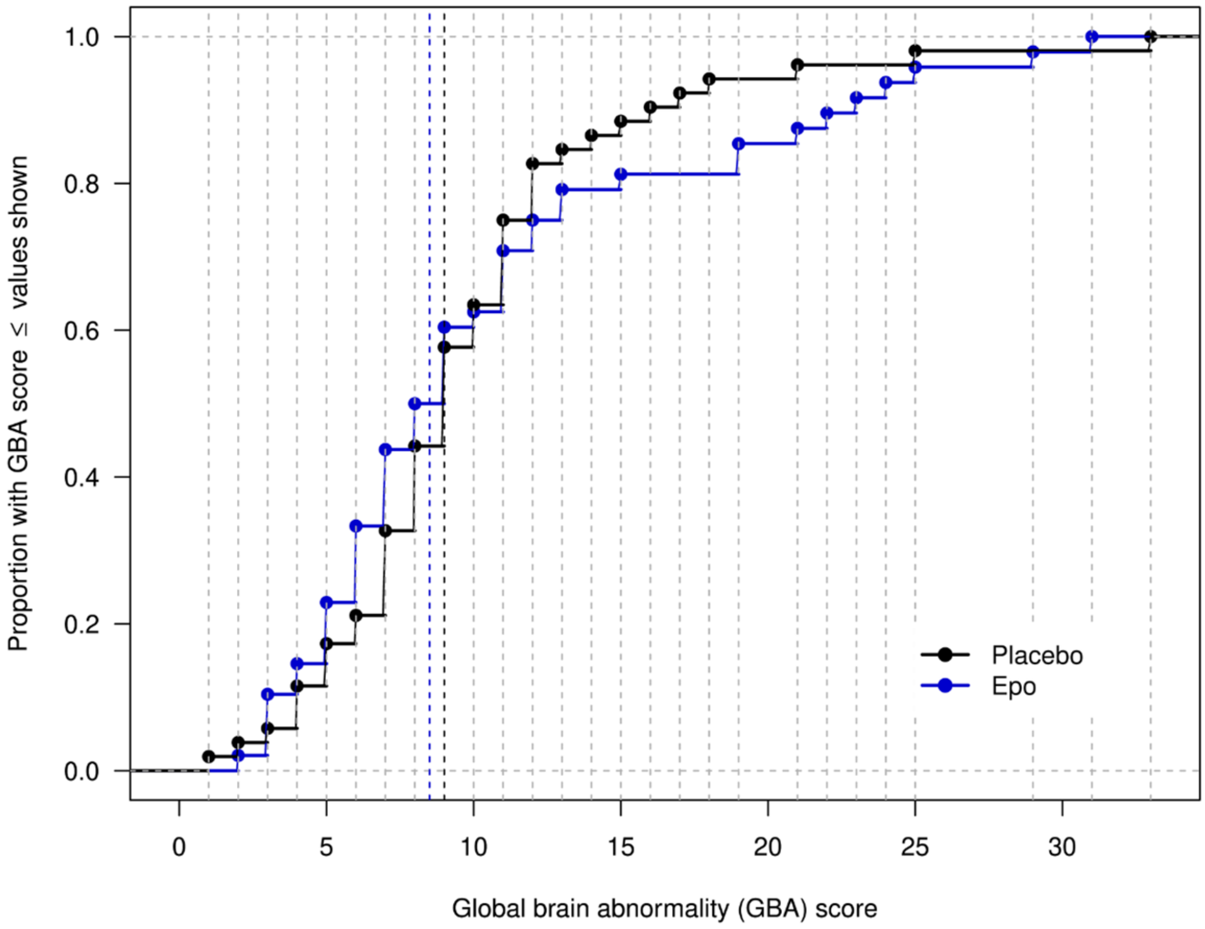

© 2022 Wellmann S et al. *JAMA Network Open.*

eFigure 4. Forest Plot of Odds Ratio (OR) Estimates (Boxes) and 95% Confidence Intervals (Lines) of the Erythropoietin (Epo) vs Placebo Treatment Effect on All Binary Secondary Outcomes Shown in the eTable of the Supplement File. Note that because all outcomes are adverse outcomes, an OR < 1 favors erythropoietin and an OR > 1 favors placebo.

Odds Ratio (OR) Epo vs. Placebo

Outcome	Total (Pla)	Events (Pla)	Total (Epo)	Events (Epo)	OR
Mod./sev. cortical grey matter abnormality	51	27	47	20	0.66
Mod./sev. deep grey matter abnormality	52	7	48	6	0.93
Mod./sev. cerebellar abnormality	52	22	47	19	0.92
PHH with drainage DOL 28	61	9	60	11	1.30
PHH with drainage PMA 36	61	8	60	11	1.49
Culture–proven infection	61	15	60	14	0.92
NEC or FIP	61	7	60	12	1.93
Supplemental oxygen at PMA 36	60	26	55	18	0.60
ROP stage 2 or higher	55	27	50	23	0.91
Periventricular leukomalacia at PMA 36	58	12	50	14	1.50
Death up to TEA	61	5	60	10	2.24

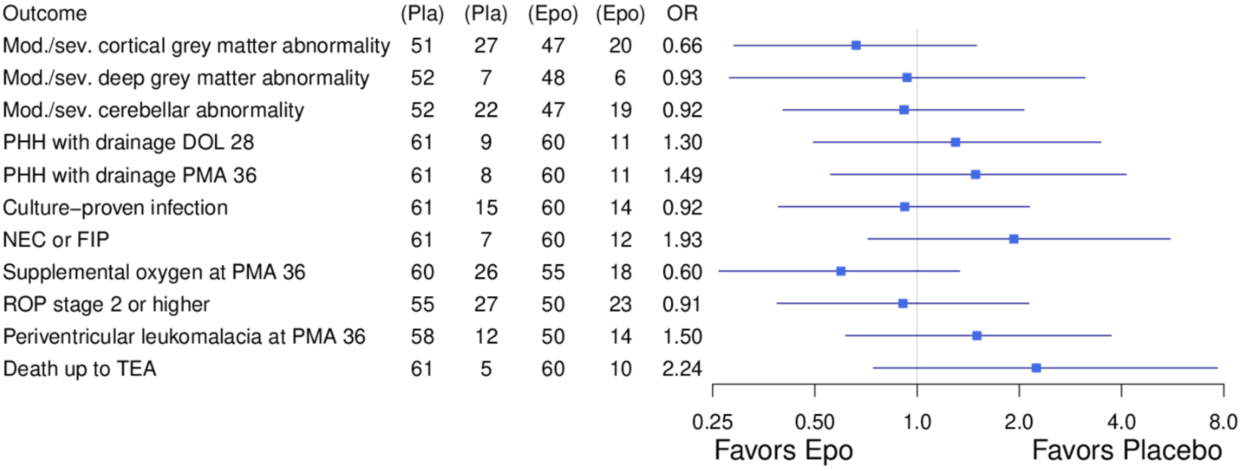

© 2022 Wellmann S et al. *JAMA Network Open.*
