# Supplementary material for: Safety and Short-term Outcomes of High-Dose Erythropoietin in Preterm Infants With Intraventricular Hemorrhage: The EpoRepair Randomized Clinical Trial
Source: JAMA Netw Open. 2022 Dec 2;5(12):e2244744. doi: 10.1001/jamanetworkopen.2022.44744 (PMC9719050; doi:10.1001/jamanetworkopen.2022.44744)
Supplement: Supplement 3. — Nonauthor Collaborators [file jamanetwopen-e2244744-s003.pdf]

Supplemental Online Content: Nonauthor Collaborators
*First name, last name, and suffix (if applicable) are required and will appear in PubMed.

*Group Name(s): Erythropoietin for the Repair of Cerebral Injury in Very Preterm Infants (EpoRepair) Investigators							
***First Name and Middle Initial(s)**	***Last Name**	***Suffix (eg, Jr, III)**	Academic Degrees	Institution	Location (city, state/province, country)	Role or Contribution, eg, chair, principal investigator	Group (if more than 1 Group listed in the byline) and/or Subgroup (eg, Steering Committee)
Philipp	Meyer		MD	Kantonsspital Aarau	Aarau, Switzerland	Local PI	
Gabriel	Konetzny		MD	Kantonsspital Aarau	Aarau, Switzerland	Co-PI	
Corinne	Däster		MD	Kantonsspital Aarau	Aarau, Switzerland	Co-PI	
Sven M	Schulzke		MD	University Children's Hospital Basel (UKBB)	Basel, Switzerland	Local PI	
Severin	Kasser		MD	University Children's Hospital Basel (UKBB)	Basel, Switzerland	Co-PI	
Roland	Gerull		MD	University Children's Hospital Basel (UKBB)	Basel, Switzerland	Co-PI	
Mathias	Nelle		MD	Inselspital, Bern University Hospital	Bern, Switzerland	Local PI	
Benedikt	Bubl		MD	Inselspital, Bern University Hospital	Bern, Switzerland	Co-PI	
Walter	Bär		MD	Kantonsspital Chur	Chur, Switzerland	Local PI	
Brigitte	Scharrer		MD	Kantonsspital Chur	Chur, Switzerland	Co-PI	
Thomas	Riedel		MD	Kantonsspital Chur	Chur, Switzerland	Co-PI	
Jean-François	Tolsa		MD	University Center Hospital and University of Lausanne	Lausanne, Switzerland	Local PI	
Bjarte	Rogdo		MD	Kantonsspital St. Gallen	St. Gallen, Switzerland	Local PI	
Irene	Hoigne		MD	Kantonsspital St. Gallen	St. Gallen, Switzerland	Co-PI	
André	Birkenmaier		MD	Kantonsspital St. Gallen	St. Gallen, Switzerland	Co-PI	
Giancarlo	Natalucci		MD	University Hospital Zurich	Zurich, Switzerland	Co-PI	
Angelika	Berger		MD	Medical University of Vienna	Vienna, Austria	Local PI	
Alexandra	Thajer		MD	Medical University of Vienna	Vienna, Austria	Co-PI	
