# Supplementary material for: Safety and Short-term Outcomes of High-Dose Erythropoietin in Preterm Infants With Intraventricular Hemorrhage: The EpoRepair Randomized Clinical Trial
Source: JAMA Netw Open. 2022 Dec 2;5(12):e2244744. doi: 10.1001/jamanetworkopen.2022.44744 (PMC9719050; doi:10.1001/jamanetworkopen.2022.44744)
Supplement: Supplement 4. — Data Sharing Statement [file jamanetwopen-e2244744-s004.pdf]

Data Sharing Statement

Wellmann. Safety and Short-term Outcomes of High-Dose Erythropoietin in Preterm Infants With Intraventricular Hemorrhage. *JAMA Netw Open*. Published December 02, 2022. doi:10.1001/jamanetworkopen.2022.44744

Data
Data available: Yes
Data types: Deidentified participant data
How to access data: On request to the PI of the trial, sven.wellmann@gmail.com
When available: beginning date: 06-01-2024

Supporting Documents
Document types: None

Additional Information
Who can access the data: researchers whose proposed use of the data has been approved
Types of analyses: systematic review and meta-analysis
Mechanisms of data availability: with a signed data access agreement
Any additional restrictions: none
